# A simple and cost-effective method for the synthesis of strontium molybdate (SrMoO_4_) nanopowders and their application as efficient heterogeneous catalysts for Knoevenagel condensation

**DOI:** 10.1039/d6ra06036f

**Published:** 2026-08-10

**Authors:** Yousra Taoudi, Aziz Arzine, Houda Serrar, Fahd T. Al-Wadaani, Hicham Oudghiri Hassani, Souad Rakass, Khalil Azzaoui, Belkheir Hammouti, Lamy Mamdoh Mohamed Hamed, Ahmed El-Harairy, Mohammed Lachkar

**Affiliations:** a Engineering Laboratory of Organometallic, Molecular Materials and Environment (LIMOME), Faculty of Sciences, Chemistry Department, Sidi Mohamed Ben Abdellah University Po. Box 1796 (Atlas) 30000 Fez Morocco oudghiri_hassani_hicham@yahoo.com; b Laboratory of Organic Chemistry, Catalysis and Environment, Department of Chemistry, Faculty of Science, Ibn Tofaïl University Kenitra Morocco; c Chemistry Department, College of Science, Taibah University PO Box 344 Madinah Saudi Arabia; d Laboratory of Applied Organic Chemistry (LCOA), Chemistry Department, Faculty of Sciences and Techniques, Sidi Mohamed Ben Abdellah University Po. Box 2202, Imouzzer Road 30000 Fez Morocco; e Euro-Mediterranean University of Fes BP 15 Fes 30070 Morocco; f Department of Environment and Agricultural Natural Resources, College of Agricultural and Food Sciences, King Faisal University Al-Ahsa Saudi Arabia lamy.hamed@kfu.edu.sa; g Department of Chemical and Biomolecular Engineering, College of Engineering, University of Nebraska-Lincoln Lincoln NE 68588 USA ael-harairy2@nebraska.edu; h Department of Soils and Water, Faculty of Agriculture, Damietta University New Damietta 34517 Egypt

## Abstract

Strontium molybdate nanopowders, SrMoO_4_, were synthesized by calcination of an oxalate complex at 700 °C. The oxalate precursor was characterized by Fourier-transform infrared (FTIR) spectroscopy and thermogravimetric analysis (TGA). The synthesized strontium molybdate was subsequently analyzed by X-ray powder diffraction (XRD), Raman spectroscopy (RS), Scanning electron microscopy (SEM), and Energy-dispersive X-ray spectroscopy (EDX). The synthesized materials were evaluated for their catalytic performance in the Knoevenagel condensation of different aromatic aldehydes with malononitrile at room temperature, and the results were compared.

## Abstract 2

Strontium molybdate nanopowders, SrMoO_4_, were synthesized by calcination of an oxalate complex at 700 °C.

## Graphical abstract

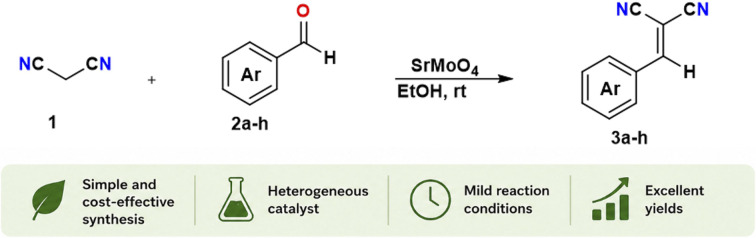

## 1 Introduction

The Knoevenagel condensation reaction is important in organic synthesis. It forms a new carbon–carbon (C–C) bond by reacting an aldehyde or ketone with a compound possessing an active methylene group, such as malonic or cyanoacetic esters. This reaction, catalyzed by a weak base, leads to α,β-unsaturated carbonyl derivatives formed through the elimination of a water molecule.^1–8^ It is widely utilized for the synthesis of diverse heterocyclic compounds through multicomponent reactions. It is employed in the preparation of various heterocyclic systems through multicomponent reactions. It has numerous applications in various fields, including pharmaceuticals, perfumes, and cosmetics,^9–11^ as well as herbicides, insecticides, and polymers.^12–25^ Most notably, this reaction serves as an important step in the preparation of numerous biologically active natural products. These natural products serve as a major source for drug discovery and development, offering therapeutic potential against a broad spectrum of diseases.^26–28^ Nowadays, nanomaterials play a crucial role in heterogeneous catalytic applications due to their high surface activity, tunable pore sizes, and ease of surface modification.^29–33^ Ternary molybdate-based scheelite-type metal oxides (A^2+^B^6+^O_4_) have already been explored in catalysis and related applications. Due to their relatively low calcination temperatures below 1200 °C and the higher flexibility for ionic substitution at both the A and B sites, these materials are attracting significant attention for use in low-temperature co-fired technology.^34,35^

Strontium molybdate (SrMoO_4_) is one of the materials in this class that crystallizes in the Scheelite structure. In SrMoO_4_, the Sr^2+^ cation occupies sites coordinated by eight O^2−^ ions, while the Mo^6+^ cation is surrounded by four O^2−^ ions in a tetrahedral coordination environment. This material has been extensively investigated for its application in electronic devices,^36^ luminescence,^37^ electrochemical hydrogen evolution reaction,^38^ optical and dielectric properties.^39^ SrMoO_4_ has been synthesized using various methods, including microwave-assisted hydrothermal,^40^ co-precipitation,^41^ solvothermal,^42^ hydrothermal,^43^ and sol–gel.^44^ These methods often require solvents, high processing temperatures, long reaction times, or multiple preparation steps.

The primary objective of the present work is to synthesize SrMoO_4_ nanoparticles employing a novel catalyst synthesis method. In fact, this work introduces a simple solvent-free solid-state synthesis based on the direct reaction of strontium nitrate, ammonium molybdate, and oxalic acid. This approach enables the formation of SrMoO_4_ nanoparticles at relatively low temperatures *via* a simple reaction method, thereby reducing both synthesis time and energy consumption while eliminating the use of solvents. The obtained nanoparticles exhibit high phase purity, highlighting the effectiveness of the proposed synthesis strategy. The synthesized catalyst was analyzed by several characterization methods, including FTIR, TGA, RS, XRD, EDX, and SEM. In addition, its catalytic performance was investigated through the Knoevenagel condensation reaction. Thus, a study of various parameters was conducted to identify the optimal reaction conditions, enabling the production of the desired products with excellent yields.

## 2 Materials and methods

### 2.1 Preparation of SrMoO_4_

General procedure for the preparation of the SrMoO_4_ catalyst:

SrMoO_4_ nanoparticles were synthesized by a simple operating method based on the thermal decomposition of an oxalate precursor through a solid-state synthesis process of oxalic acid (H_2_C_2_O_4_·2H_2_O), ammonium molybdate ((NH_4_)_6_Mo_7_O_24_ 4H_2_O), and strontium nitrate (Sr(NO_3_)_2_) in the molar ratio 4/1/1, respectively (all reagents were obtained from Sigma-Aldrich). The mixture was thoroughly ground and heated at 150 °C for 60 min. The obtained nanopowders were then dried at 170 °C, followed by calcination at 700 °C for 2 h. The calcined nanoparticles were subsequently examined using different characterization techniques, such as SEM-EDX, XRD, and Raman spectroscopy.

### 2.2 Material characterization

The synthesized strontium molybdate oxalate complex was analyzed by thermogravimetric analysis using a LINSEIS STA PT1600 thermal analyzer. Fourier-transform infrared spectra were recorded with a BRUKER INVENIO-S spectrometer over the 400–4000 cm^−1^ spectral range. The structural properties of the SrMoO_4_ nanopowders were analyzed by X-ray diffraction using a PANalytical X'Pert Pro diffractometer equipped with Cu-Kα radiation (*λ* = 1.54060 Å). The measurements were performed at an operating voltage of 40 kV and a current of 30 mA, with data collected over a 2*θ* range of 10–80°. Raman measurements were performed using a Horiba Scientific XploRa PLUS Raman microscope with a 532 nm laser as the excitation source. The spectra were collected in the wavenumber range from 50 to 1000 cm^−1^. Morphological and elemental analyses were carried out utilizing a JEOL JSM-IT500HR scanning electron microscope coupled with energy-dispersive X-ray spectroscopy. The instrument was operated with a probe current ranging from 10 pA to 20 nA and an acceleration voltage between 0.5 and 30 kV.

### 2.3 Typical procedure for the Knoevenagel condensation reaction

First, 1 mmol of aromatic aldehyde was mixed in 1 mL of ethanol in the presence of the SrMoO_4_ nanocatalyst. The resulting mixture was maintained under stirring at room temperature, and the progress of the reaction was followed by TLC. After the appearance of a precipitate, ethanol was added to facilitate product recovery and catalyst removal. The reaction mixture was subsequently filtered to separate the catalyst, and the resulting filtrate was allowed to cool, affording the pure products. All yields reported in this work correspond to isolated yields obtained after this purification procedure. The obtained compounds were verified by ^1^H NMR and ^13^C NMR analyses (SI Section 2). The melting points of each compound were also determined and compared with literature values, showing good agreement and thus confirming the structures of the synthesized compounds.

## 3 Results and discussion

### 3.1 Characterizations of the strontium molybdate oxalate complex

**Fig. 1:**
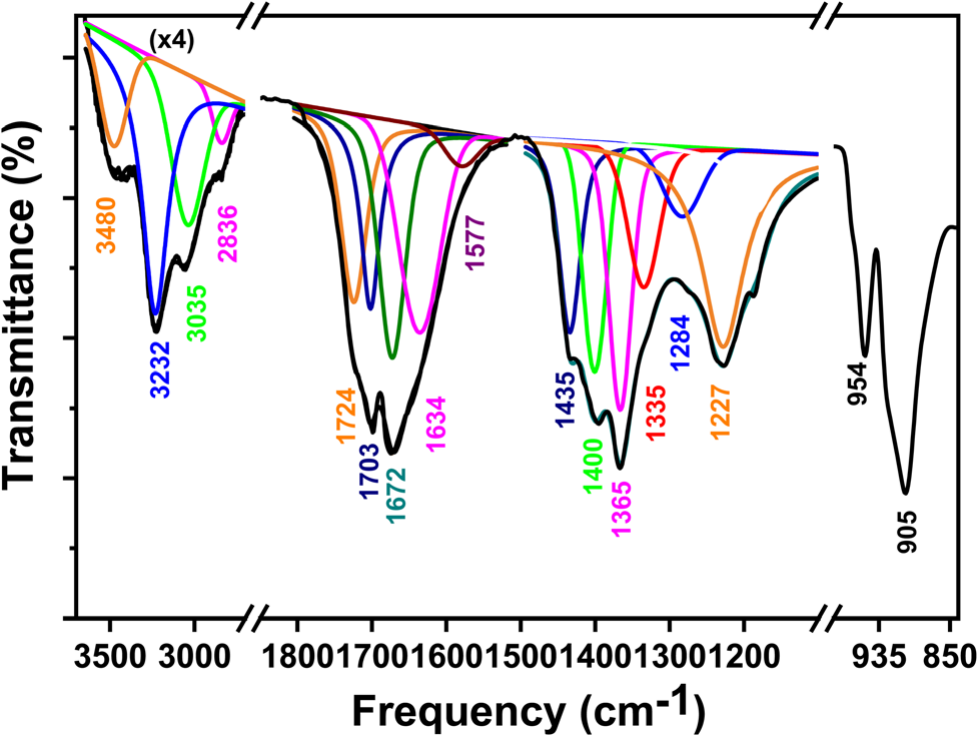
Fourier transform infrared (FTIR) spectrum of the strontium molybdate oxalate complex.

The functional groups present in the strontium oxalate complex were characterized by FTIR spectroscopy (Fig. 1). The spectrum exhibits two broad absorption bands, located in the 3614–2700 cm^−1^ and 1800–1510 cm^−1^ regions. Deconvolution reveals several distinct bands. Indeed, the spectrum shows absorption bands at 1703 and 1724 cm^−1^, corresponding to the C=O stretching modes of the oxalate group,^45,46^ whereas the band observed at 1634 cm^−1^ is attributed to the H_2_O bending vibration.^47–49^ The 1400 cm^−1^ band confirms C–O stretching. Meanwhile, the bands appearing at 1284 and 1335 cm^−1^ are attributed to *δ*(O–C–O) and *ν*(C–O), respectively.^50,51^ The spectrum also exhibits bands at 954 and 905 cm^−1^, assigned to the stretching vibrations of Mo=O bands.^52^ Moreover, the presence of ammonia (NH_3_) and ammonium (NH_4_^+^) species is evidenced by their asymmetric and symmetric deformation modes, observed at 1227 cm^−1^ (*δ*_as_(NH_3_)) and 1577 cm^−1^ (*δ*_s_(NH_3_)), as well as at 1435 cm^−1^ (*δ*_as_(NH_4_^+^)) and 1672 cm^−1^

(*δ*_s_(NH_4_^+^)), respectively.^48,50,53,54^ The band at 2836 cm^−1^ indicates the existence of ammonium ions, while the bands located at 3035 and 3232 cm^−1^ confirm the presence of coordinated ammonia. The absorption band located at 3480 cm^−1^ is attributed to the O–H bridging group linking two metal ions.^51–54^ In addition, the band observed at 1365 cm^−1^ is attributed to the *δ*(OH) bending vibration.^49^ These finding confirm the occurrence of functional groups associated with NH_4_^+^ ions, NH_3_, oxalate, hydroxyl (–OH), water, and oxo (Mo=O) species, in the strontium oxalate prepared precursor.

**Fig. 2:**
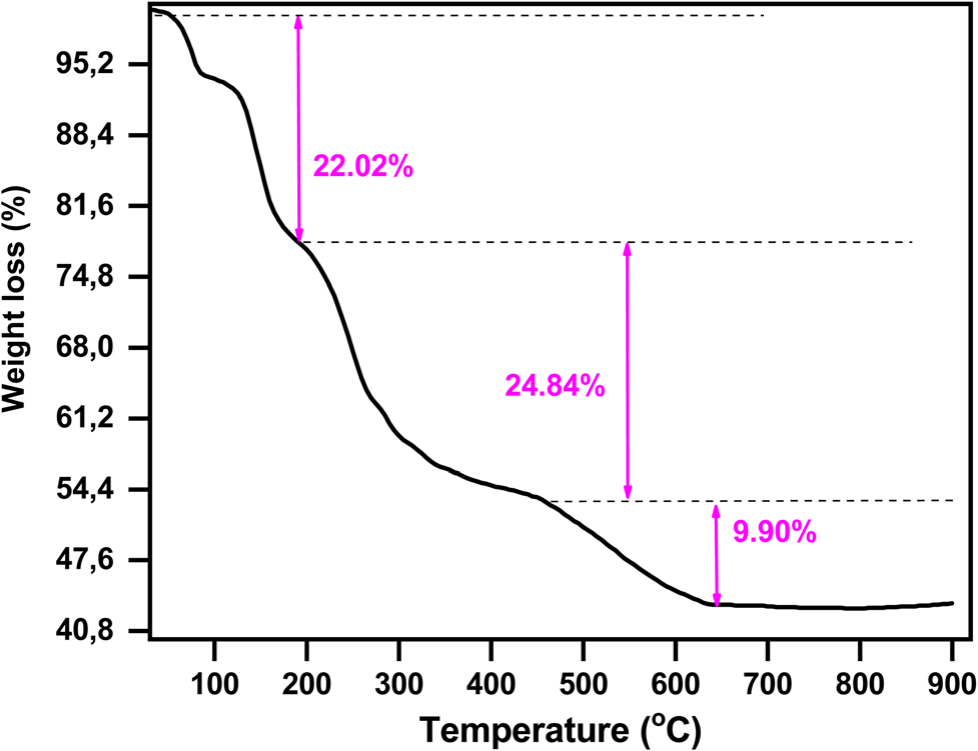
Thermogravimetric analysis (TGA) of the strontium molybdate oxalate complex.

Fig. 2 presents the thermogravimetric (TG) curve of the prepared complex, revealing three distinct decomposition stages.

The first stage (25–185 °C) shows a weight loss of 22.02%, attributed to the release of physically adsorbed and coordinated water molecules. The second stage (185–454 °C), with a weight loss of 24.84%, corresponds to the complete decomposition of the oxalate precursor. The final stage (454–630 °C) exhibits a weight loss of 9.90%, associated with the elimination of hydroxyl (–OH) groups.

Based on an examination of the possible oxidation states of molybdenum and strontium, as well as data obtained by FTIR and TGA, the composition of the precursor prepared can be proposed as follows: (NH_3_)(NH_4_^+^)_2_SrMoO_2_(C_2_O_4_)_1.5_(OH)_3_·7H_2_O

It should be noted that the total weight loss measured, of 56.76% up to 700 °C, corresponds exactly to the calculated value of 57.15%, which confirms the validity of the proposed chemical formula.

### 3.2 Strontium molybdate characterization

#### 3.2.1 X-ray diffraction analysis

**Fig. 3:**
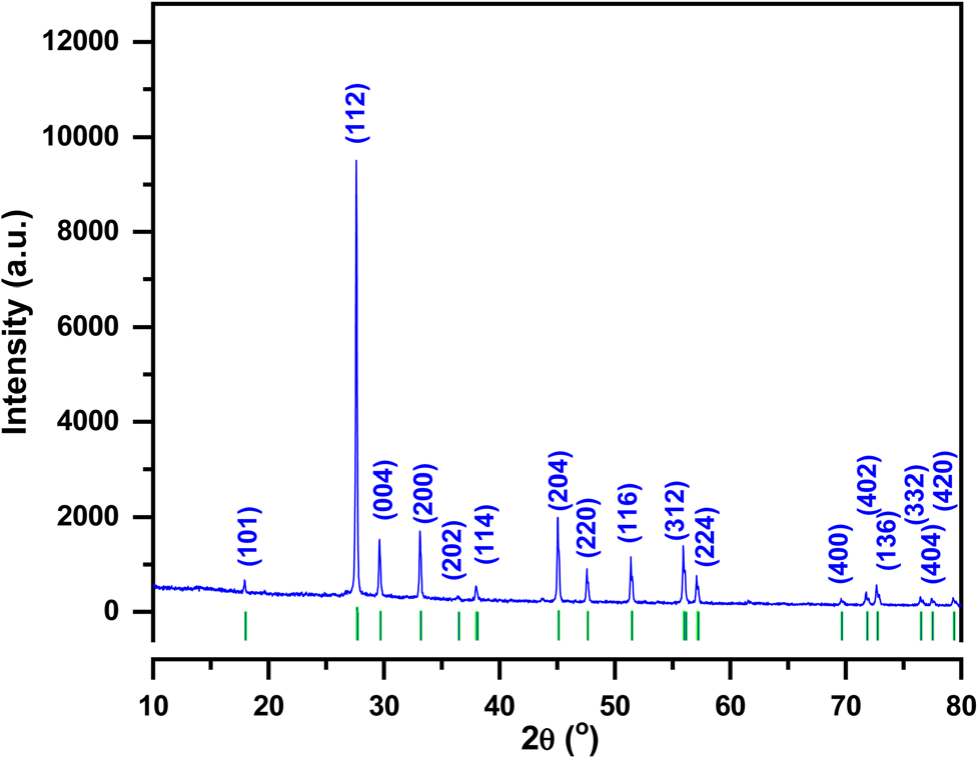
X-ray diffraction pattern of the synthesized SrMoO_4_ nanopowders.

The X-ray diffraction (XRD) patterns of SrMoO_4_ synthesized through a novel solid-state reaction are presented in Fig. 3. All diffraction peaks were indexed to the tetragonal scheelite-type crystal structure of SrMoO_4_, consistent with the JCPDS card no. 08-0482, with no detectable impurity phases.^55^

The Debye–Scherrer equation (eqn (1)) was employed to determine the average crystallite size (*D*) using the strongest diffraction peak associated with the (112) plane.1
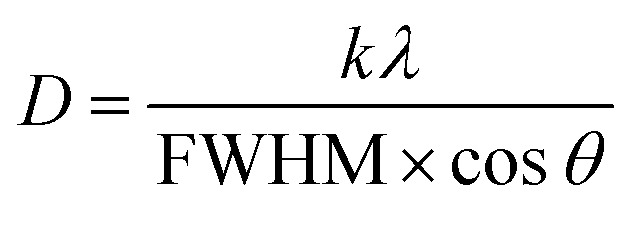
where *k* = 0.9 is the shape factor, *θ* represents the Bragg diffraction angle, *λ* denotes the X-ray wavelength, and FWHM corresponds to full width at half maximum of the diffraction peak.^56–59^

The calculated crystallite size was approximately 81 nm after calcination at 700 °C.

#### 3.2.2 Raman spectroscopy

**Fig. 4:**
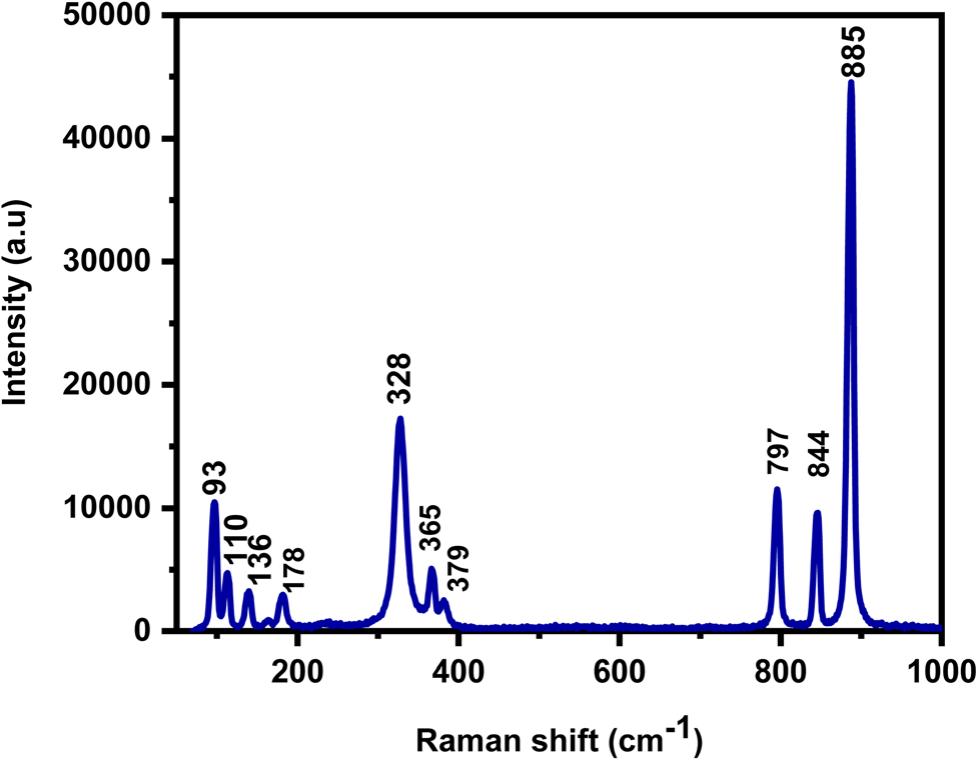
Raman spectrum of the prepared SrMoO_4_.

The Raman spectrum of the synthesized SrMoO_4_ calcined at 700 °C, recorded in the range of 50–1000 cm^−1^, is presented in Fig. 4. Group theory analysis predicts 26 different vibrational modes for MMoO_4_ compounds, which can be expressed as follows:*r* (Raman + Infrared) = 3*A*_g_ + 5*B*_g_ + 5*E*_g_ + 5*A*_u_ + 3*B*_u_ + 5*E*_u_where the *A*_g_, *B*_g_, and *E*_g_ vibrations correspond to Raman-active modes. The B mode is non-degenerate, while the *E* mode is doubly degenerate. The *A*_u_ and *E*_u_ modes are associated with zero-frequency acoustic vibrations, whereas the others correspond to vibrational modes. Moreover, the *A*_g_, *B*_g_, and *E*_g_ modes arise from the same type of atomic motions observed in the SrMoO_4_ crystal structure. Consequently, 13 Raman-active modes are expected for SrMoO_4,_ as indicated in eqn (2).^60^

2 *r* = 3*A*_g_ + 5*B*_g_ + 5*E*_g_

Raman vibrational modes are generally classified into two principal categories: external modes and internal modes. Modes below 328 cm^−1^ represent external vibrations, while internal modes are present in the region of higher wave numbers. As illustrated in Fig. 4, the intense Raman band observed at 885 cm^−1^ is assigned to the symmetric stretching mode *Ѵ*_1_(*A*_g_) of the tetrahedral [MoO_4_]^2−^ group in the crystal structure of SrMoO_4_. Raman bands observed at 844 and 797 cm^−1^ are assigned to the antisymmetric stretching vibration modes *Ѵ*_3_(*B*_g_) and *Ѵ*_3_(*E*_g_), respectively. In addition, the peaks appearing at 379, 365, and 328 cm^−1^ are related to the antisymmetric and symmetric bending vibration modes *Ѵ*_4_(*E*_g_), *Ѵ*_4_(*B*_g_), and *Ѵ*_2_(*A*_g_), respectively. The free rotational mode was identified at 178 cm^−1^, and the external vibration modes were observed in the 93–136 cm^−1^ range. These observations are in good agreement with the values as shown in the literature.^61–69^

#### 3.2.3 Scanning electron microscope, elemental mapping, and EDX analysis

**Fig. 5:**
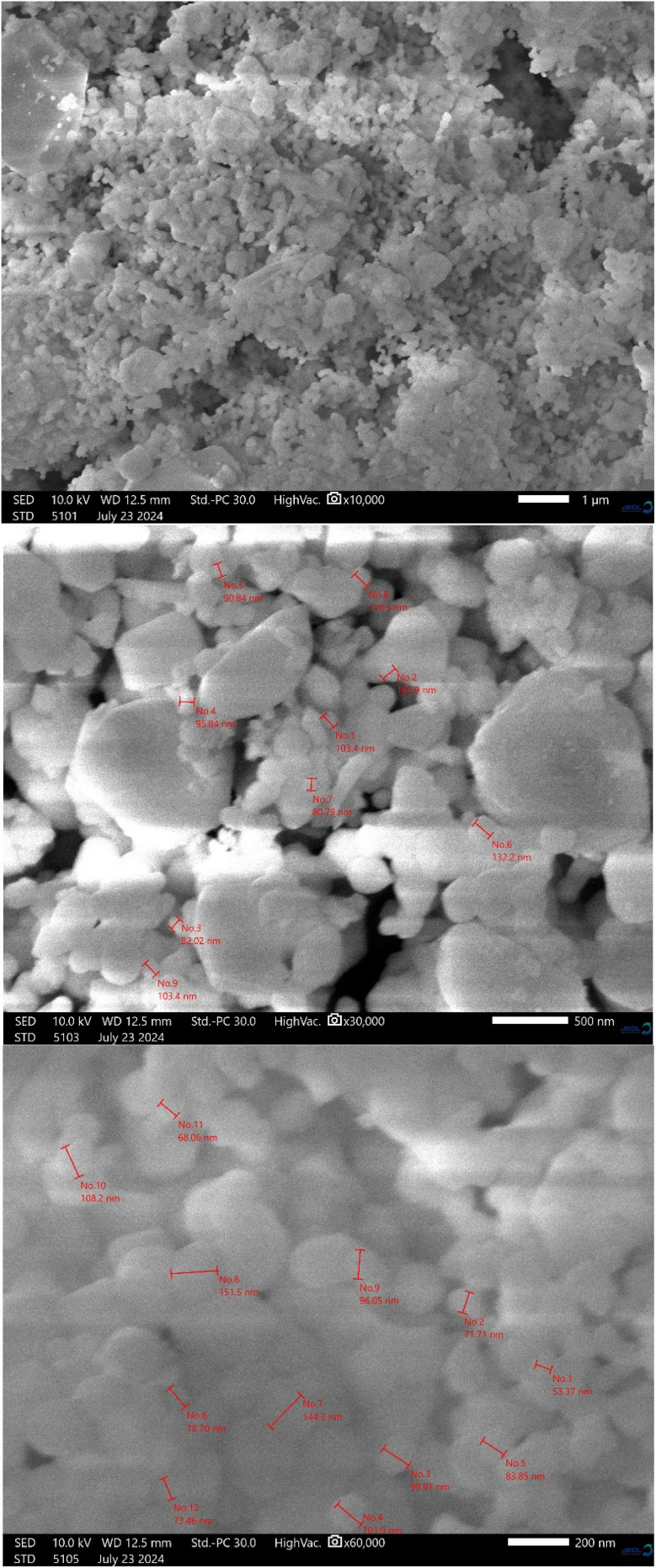
Scanning electron microscope images of SrMoO_4_ nanoparticles synthesized at 700 °C.

**Fig. 6:**
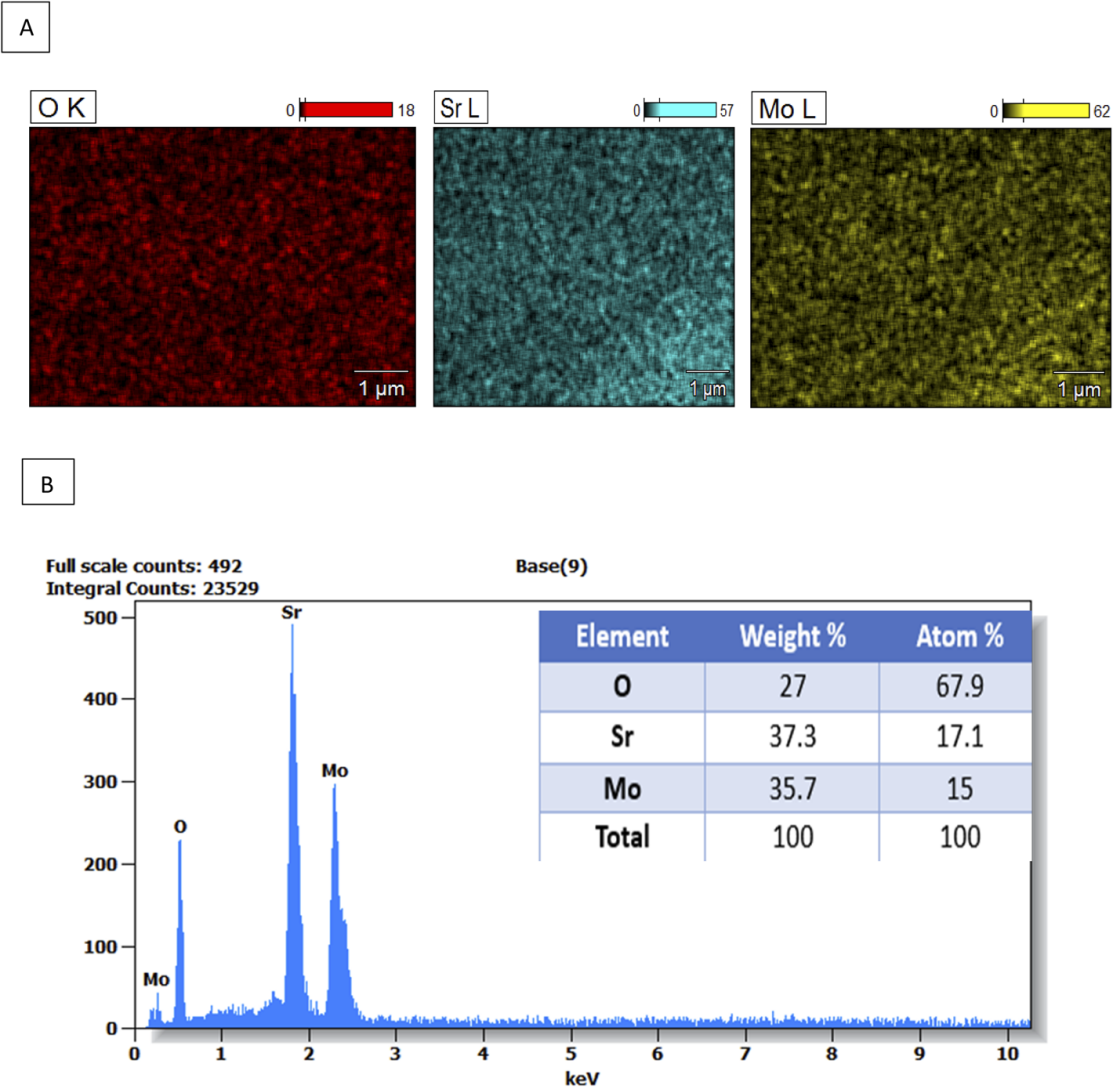
(A) Elemental mapping, and (B) EDX spectrum of SrMoO_4_.

The SEM images of SrMoO_4_ synthesized by the solid-state process after heat treatment at 700 °C for 2 hours are shown in Fig. 5. The sample obtained at 700 °C consists of uniform spherical particles (Fig. 5). Elemental mapping and EDX spectroscopy were performed to analyze the distribution of elements on the particle surfaces, as shown in Fig. 6A and B. The results indicate a relatively homogeneous distribution of O, Mo, and Sr across the sample surface. These results are consistent with the XRD analysis results.

### 3.3 Knoevenagel condensation reactions

**Scheme 1:**
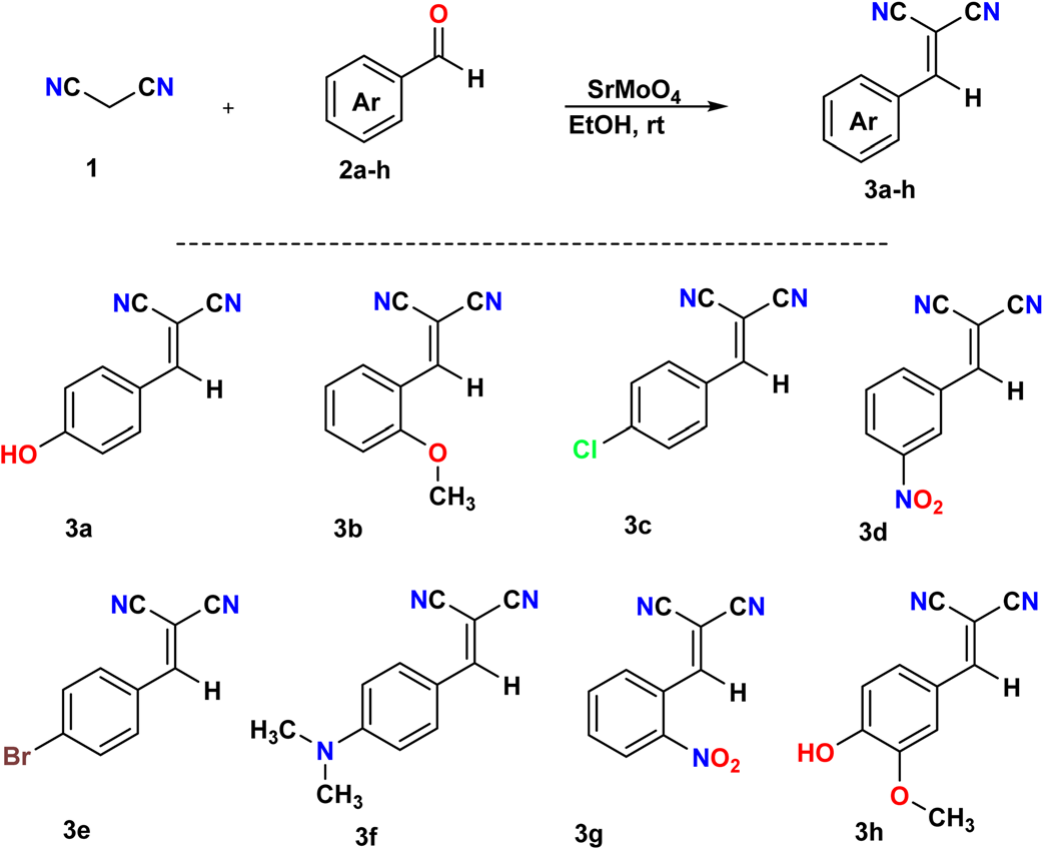
Knoevenagel reaction of aldehydes with malononitrile in the presence of the SrMoO_4_ nanocatalyst.

The heterogeneous catalytic activity of the prepared SrMoO_4_ nanopowder was evaluated in the Knoevenagel condensation reaction using benzaldehyde and malononitrile as test models (Scheme 1). To determine the optimal reaction conditions, two key parameters, namely catalyst amount and solvent, were systematically investigated, and the results are summarized in Table 1.

**Table 1:** Effect of solvent and catalyst amount on the Knoevenagel reaction

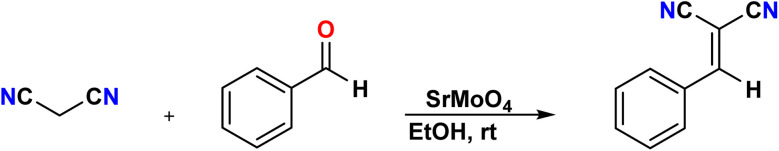				
Effect of solvent				
Entry	Catalyst	Catalyst amount (g)	Solvent	Yield (%)
1	SrMoO_4_	0.010	EtOH	75%
2	SrMoO_4_	0.010	CyH	49%
3	SrMoO_4_	0.010	CHCl_3_	64%
4	SrMoO_4_	0.010	EtOAc	44%

**Effect of catalyst mass**				
5	Neat	—	EtOH	23%
6	SrMoO_4_	0.005	EtOH	83%
7	SrMoO_4_	0.010	EtOH	86%
8	SrMoO_4_	0.020	EtOH	95%
9	SrMoO_4_	0.030	EtOH	92%

Firstly, the effect of different solvents, namely cyclohexane (CyH), chloroform (CHCl_3_), ethyl acetate (EtOAc), and ethanol (EtOH), was investigated (Table 1, entries 1–4). The results showed that the highest yield (75%) was obtained when ethanol (EtOH) was used as the solvent. This result can likely be explained by the polar protic nature of ethanol, which promotes greater interaction between the reactants and the catalyst surface, thereby facilitating product formation. Ethanol may also enhance catalyst dispersion and improve the solubility of the reactants compared with the other solvents tested.

The effect of catalyst mass was also examined (Table 1, entries 5–9). In the absence of the SrMoO_4_ catalyst, only 23% of the desired product was obtained. However, increasing the catalyst amount from 0.005 to 0.020 g led to a significant improvement in yield, reaching a maximum of 95% (Table 1, entry 8). This enhancement can be due to the increased number of available active sites. However, a further increase to 0.030 g resulted in a slight decrease in yield to 92% (Table 1, entry 9), which was probably due to catalyst aggregation. Therefore, 0.020 g of SrMoO_4_ nanocatalyst was identified as the optimal catalyst mass (Table 1, entry 8).

Accordingly, the optimal conditions were selected as follows: 0.02 g of SrMoO_4_ nanocatalyst and 1 mL of ethanol (EtOH) as solvent. Hence, this result demonstrates the high catalytic efficiency of the synthesized strontium molybdate in the Knoevenagel condensation reaction compared to previous research works found in the literature, as presented in Table 2. As shown in this table, the present catalyst exhibits catalytic activity comparable to, and even superior to, that of many previously reported systems, achieving a high yield (95%) in a short reaction time (0.15 hours) under mild conditions, using ethanol as an environmentally friendly solvent at room temperature. Compared to previously reported catalysts, the SrMoO_4_ nanocatalyst combines ease of use, environmentally friendly reaction conditions, and excellent catalytic efficiency, highlighting its potential as an effective heterogeneous catalyst for Knoevenagel condensation reactions.

**Table 2:** Comparison of the catalytic performance of the present SrMoO_4_ nanocatalyst with previously reported heterogeneous catalysts for the Knoevenagel condensation.^70–72^

CP's metal center(s)	Catalyst load (% mol)	Solvent	Temperature (°C)	Time (h)	Yield (%)	Typical ligands
Co(ii) or Ni(ii)	0.19	EtOH	80	0.16	>95	Carboxylates, pyridines, other organic ligands
Zn(ii)	3	THF	40	1.5	98	Carboxylates, organic ligands, polydentate ligands
Cd(ii)	2	MeOH	r.t	1	100	Carboxylates, nitrile-containing organic ligands
Pb(ii)	9.2	Toluene	85	5	99	Carboxylates, various organic ligands
Cu(ii)	0.2	Neat	60	Not informed	>99	Organic ligands like pyridine or nitrile compounds
Tb(iii)	Not informed	ACN	60	24	99	Carboxylates, macrocycles, phosphonates
This work: Sr(ii)	2	EtOH	r.t	0.15	95	MoO_4_ (tetramolybdate(VI))

In another study, to evaluate the efficacy, substrate compatibility, and robustness of the developed protocol, various aromatic aldehydes containing either electron-donating functional groups or electron-accepting functional groups were coupled with malononitrile in the condensation reaction, and the results are summarized in (Table 3, entries 1–8).

**Table 3:** Knoevenagel condensation of aldehydes catalyzed by SrMoO_4_: reaction time, yields, and melting points

Compds	Ar	Reaction time (min)	Yield (%)	MP (°C)		References
				Obtained	Reported	
3a	4-(OH)C_6_H_4_	15	63%	184	[183–184]	73
3b	2-(OCH_3_)C_6_H_4_	10	80%	90	–a	–a
3c	4-(Cl)C_6_H_4_	10	96%	165	[163–165]	74
3d	3-(NO_2_)C_6_H_4_	10	75%	102	[103–104]	73 and 74
3e	4-(Br)C_6_H_4_	10	87%	156	[153–155]	73
3f	4-(N(CH_3_)_2_)C_6_H_3_	10	75%	178	[179–180]	73–75
3g	2-(NO_2_)C_6_H_4_	10	72%	142	[140–142]	76
3h	3-(OCH_3_)-4-(OH)C_6_H_3_	15	69%	130	[131–132]	77

a No melting point is reported in the literature; no references are available.

The results summarized in Table 3, indicate, the associated Knoevenagel products were obtained in high yields are observed varying between 63–96% for all aromatic aldehydes containing either electron-donating groups, such as methoxy (–OCH_3_), methyl (–CH_3_), Hydroxyl (–OH), and dimethylamino (–N(CH_3_)_2_), or electron-accepting groups, such as nitro (–NO_2_), chloro (–Cl), and bromo(–Br) substituents (Table 3). These results highlight the remarkable efficiency of the SrMoO_4_ nanocatalyst in promoting the reaction, demonstrating its broad scope of application and suitability for a wide variety of substrates. These results prove the good catalytic performance of the SrMoO_4_ nanocatalyst and its applicability to a broad range of aromatic aldehydes. In general, substrates bearing electron-withdrawing groups led to higher yields than those containing electron-donating groups, owing to the increased electrophilic character of the carbonyl carbon, which favors nucleophilic attack and promotes the condensation reaction.

**Scheme 2:**
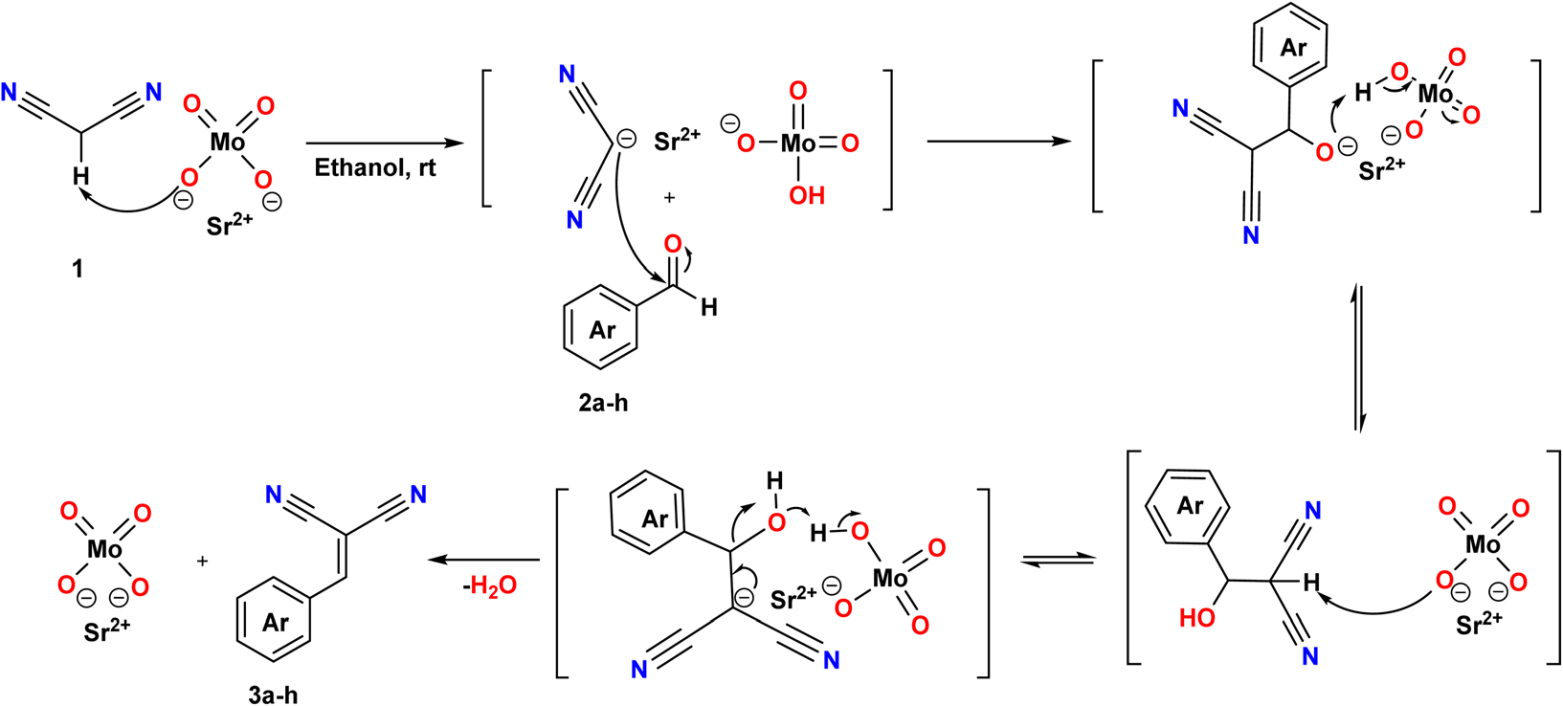
Proposed plausible catalytic mechanism for the Knoevenagel condensation using the SrMoO_4_ nanocatalyst.

Based on the obtained results, a plausible mechanism for the Knoevenagel condensation catalyzed by SrMoO_4_ is proposed, as illustrated in Scheme 2. The reaction is assumed to occur on the surface of the SrMoO_4_ nanocatalyst through a cooperative acid–base catalytic pathway. The carbonyl group of the aldehyde is activated through coordination with the Lewis acidic Sr^2+^ surface sites, which enhances the electrophilicity of the carbonyl carbon. Simultaneously, the surface oxygen atoms, acting as Lewis basic sites, promote the deprotonation of the methylene group of malononitrile, generating a reactive carbanion. The resulting nucleophilic species attacks the activated carbonyl carbon to form an intermediate, which subsequently undergoes proton transfer followed by dehydration to afford the corresponding Knoevenagel product. Finally, the product desorbs from the catalyst surface, regenerating the active sites for the next catalytic cycle. This mechanism is proposed based on the generally accepted surface acid–base properties of metal molybdates and should be regarded as a plausible catalytic pathway. On the other hand, the reaction rate is expected to be influenced by several kinetic parameters, including catalyst loading, reaction temperature, solvent nature, and the electronic properties of the aldehyde substrate. Increasing the catalyst loading enhances the number of accessible active sites, thereby accelerating the reaction until an optimum catalyst amount is reached. In addition, aldehydes bearing electron-withdrawing substituents generally react faster than those containing electron-donating groups due to the increased electrophilicity of the carbonyl carbon.^78,79^

**Fig. 7:**
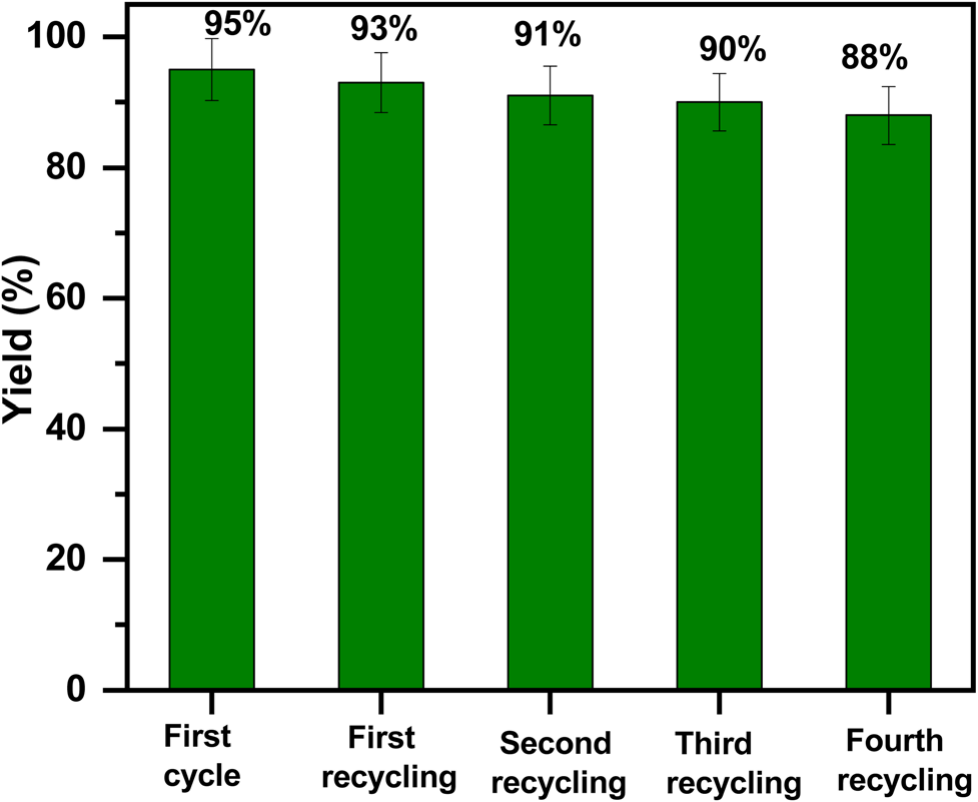
Recycled catalyst against Knoevenagel condensation reaction using benzaldehyde and malononitrile in the presence of SrMoO_4_ nanocatalysts (EtOH and *m* = 0.02 g).

On the other hand, the recyclability of the prepared heterogeneous catalyst was investigated by performing four successive cycles of the Knoevenagel condensation reaction using benzaldehyde and malononitrile under identical operating conditions (EtOH, *m*_SrMoO4_ = 0.02 g). As illustrated in Fig. 7, the catalytic performance gradually declined, decreasing from nearly 95% during the first cycle to about 88% after the fourth cycle. Such a reduction in activity can be attributed to several factors commonly observed in heterogeneous catalytic processes. First, a minor loss of catalyst during the separation and cleaning procedures may reduce the number of accessible active sites. Second, the deposition of intermediate products on the catalyst surface can impede the adsorption of reactant molecules by covering the active sites. Furthermore, repeated use may induce slight changes in the catalyst's structural and surface properties, thereby contributing to a reduction in its activity. Nevertheless, the catalyst still maintained a relatively high efficiency of approximately 88% after four cycles, demonstrating its satisfactory stability and good potential for repeated applications.

## 4 Conclusions

Strontium molybdate (SrMoO_4_) was successfully synthesized as a nanopowder using a novel, simple process. XRD, RS, SEM, and EDX analyses confirmed that the title compound possesses a scheelite-type structure and crystallizes as particles with homogeneous shapes. The prepared strontium molybdate can be used as an efficient catalyst for the Knoevenagel condensation between benzaldehydes and malononitrile, leading to the formation of substituted olefins. Various aromatic aldehydes were employed as substrates in the presence of 0.02 g of SrMoO_4_, achieving good to excellent yields (63–96%) in EtOH at room temperature.

## Author contributions

Yousra Taoudi: writing – original draft, writing – review and editing, methodology, formal analysis, investigation, conceptualization. Hicham Oudghiri Hassani: software, investigation, methodology, project administration, validation, writing – review and editing, supervision. Souad Rakass: data curation, software, investigation. Aziz Arzine: software, formal analysis. Houda Serrar: software, formal analysis. Fahd T. Al-Wadaani^:^ software, investigation, methodology. Mohammed Lachkar: supervision. Khalil Azzaoui: writing – review and editing, validation, project administration, resources. Belkheir Hammouti: formal analysis, validation. Lamy Mamdoh Mohamed Hamed, and Ahmed El-Harairy: methodology, formal analysis, writing – review and editing, supervision.

## Conflicts of interest

There are no conflicts to declare.

## Supplementary Material

RA-OLF-D6RA06036F-s001

## Data Availability

The authors declare that the data supporting the findings of this study are available within the article and its supplementary information (SI). Additional raw data, including FTIR, TGA, XRD, Raman spectroscopy, SEM-EDX analyses, and NMR spectra for all synthesized compounds, are available from the corresponding authors upon reasonable request. Supplementary information: spectroscopic data and copies of NMR ^1^H and ^13^C spectra of the synthesized compounds 3a–h, and all additional details can be found in the electronic Supplementary Material. See DOI: https://doi.org/10.1039/d6ra06036f.
