# Supplementary material for: A simple and cost-effective method for the synthesis of strontium molybdate (SrMoO4) nanopowders and their application as efficient heterogeneous catalysts for Knoevenagel condensation
Source: RSC Adv. 2026 Aug 10;16(40):46517–27. doi: 10.1039/d6ra06036f (PMC13456194; doi:10.1039/d6ra06036f)
Supplement: Supplementary file 1 [file RA-016-D6RA06036F-s001.pdf]

Supplementary Information (SI) for RSC Advances.
This journal is © The Royal Society of Chemistry 2026

Supplementary information

A Simple and Cost-Effective Method for the Synthesis of Strontium Molybdate ($SrMoO_4$) Nanopowders and Their Application as an Efficient Heterogeneous Catalyst for Knoevenagel Condensation

Yousra Taoudi[1], Aziz Arzine[1], Houda Serrar[1,2], Fahd T. Al-Wadaani[3], Hicham Oudghiri Hassani[1*], Souad Rakass[4], Khalil Azzaoui[1], Belkheir Hammouti[5], Lamy Mamdoh Mohamed Hamed[6*], Ahmed El-Harairy[7,8*], Mohammed Lachkar[1]

[1] Engineering Laboratory of Organometallic, Molecular Materials and Environment (LIMOME), Faculty of Sciences, Chemistry Department, Sidi Mohamed Ben Abdellah University, Po. Box 1796 (Atlas), 30000 Fez, Morocco;
[2] Laboratory of Organic Chemistry, Catalysis and Environment, Department of Chemistry, Faculty of Science, Ibn Tofaïl University, Kenitra, Morocco;
[3] Chemistry Department, College of Science, Taibah University, PO Box 344, Madinah, Saudi Arabia
[4] Laboratory of Applied Organic Chemistry (LCOA), Chemistry Department, Faculty of Sciences and Techniques, Sidi Mohamed Ben Abdellah University, Po. Box 2202, Imouzzer Road 30000 Fez, Morocco;
[5] Euro-Mediterranean University of Fes, Fes 30070, BP 15, Morocco.
[6] Department of Environment and Agricultural Natural Resources, College of Agricultural and Food Sciences, King Faisal University, Al-Ahsa, Saudi Arabia
[7] Department of Chemical and Biomolecular Engineering, College of Engineering, University of Nebraska-Lincoln, Lincoln, NE, 68588 USA
[8] Department of Soils and Water, Faculty of Agriculture, Damietta University, New Damietta, Damietta, 34517 Egypt

*Corresponding Author: Hicham Oudghiri Hassani (oudghiri_hassani_hicham@yahoo.com); Lamy Mamdoh Mohamed Hamed (lamy.hamed@kfu.edu.sa); Ahmed El-Harairy (ael-harairy2@nebraska.edu)

Table of contents

1. General information 2

2. Characterization data 3

2.1 Spectroscopic data of the synthesized of compounds 1-10 3

2.2 Copies of the (^{1}H, ^{13}C) NMR spectra of all compounds (1-8) 7

1. General information

All reagents and solvents were of A. R. grade. They were purchased from Sigma-Aldrich and other commercial suppliers and were used without further purification. The progress of the reactions was monitored by TLC, performed on pre-coated Merck silica gel 60 F_{254} plates. Column chromatography was carried out using Merck silica gel (70-230 mesh) and eluting with n-hexane/ether solutions. Melting points were determined using a KOFLER Bench apparatus. ^{1}H and ^{13}C NMR spectra were recorded at room temperature on a BRUKER AVANCE II 300 Ultra-Shield (300 MHz for ^{1}H and 75 MHz for ^{13}C) spectrometer using DMSO-d_6 solvents. The Jmod experiment, which was employed for the ^{13}C NMR spectra, reveals information on the number of ^{13}C signals (CH_3, CH_2, CH and Cq). CH_3 and CH carbons are represented by negative signals in these spectra, while CH_2 and C quaternary carbons are represented by positive signals. ^{1}H and ^{13}C NMR chemical shifts (δ) are expressed in ppm referenced to the residual proton signals in the solvent used, and proton-proton coupling constants (*J*) are reported in Hz. The spin multiplicities are reported as singlet (s), doublet (d), triplet (t), multiplet (m), doublet of doublets (dd), doublet of quadruplet (dq), triplet of doublets (td), doublet of triplets (dt) and broad (br).

2. Characterization data

2.1Spectroscopic data of the synthesized of compounds 1-10

***2-(4-hydroxybenzylidene)malononitrile* 1:**

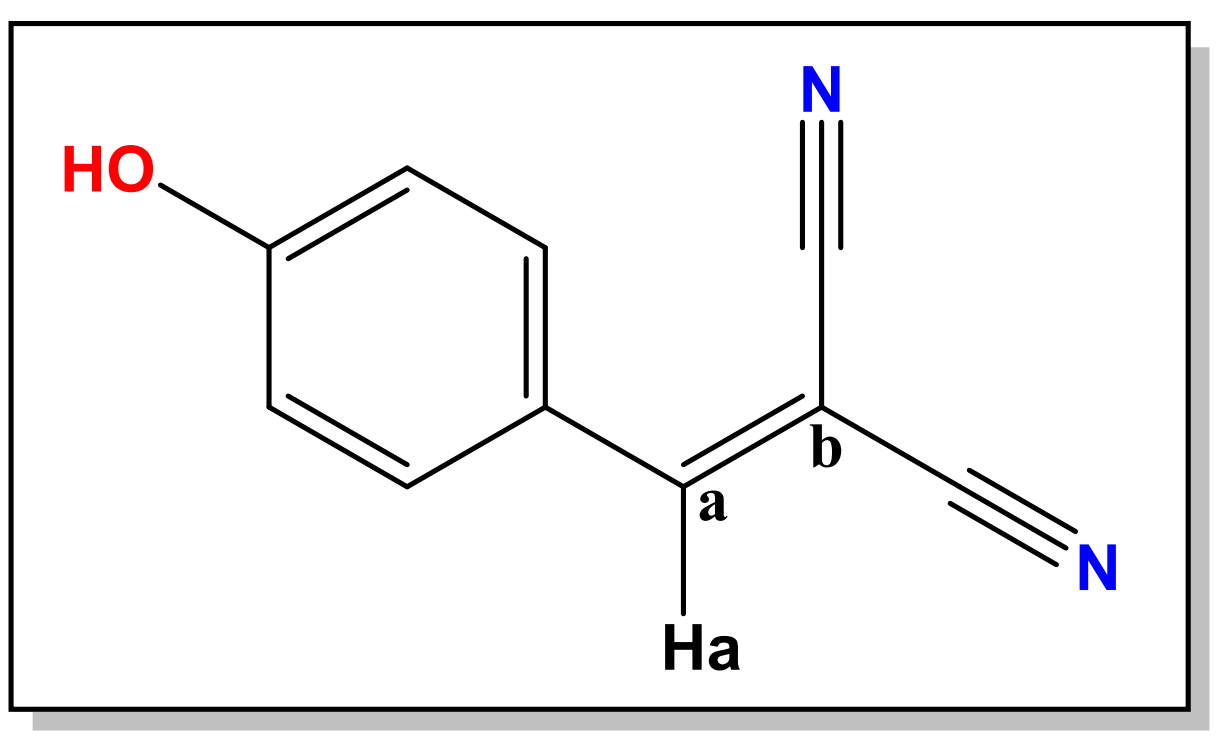

Pale yellow solid; **Yield** = 63%; **m.p.**: 184-186 °C.

^{1}H NMR (300 MHz, DMSO-d_6) (δ in ppm): 6.97 (d, 2H, *J* = 11.52 Hz, Ar–H), 6.89 (d, 2H, *J* = 8.85 Hz, Ar–H), 8.31 (s, 1H, Ha), 11.02 (s, 1H, OH),

^{13}C NMR (75 MHz, DMSO-d_6) (δ in ppm): 75,48 (C_b), 114.66, 115.57, 117.11, 123.21, 134.34, 160.95 (C-OH), 164.44 (C_a).

***2-(2-methoxybenzylidene)malononitrile* 2:**

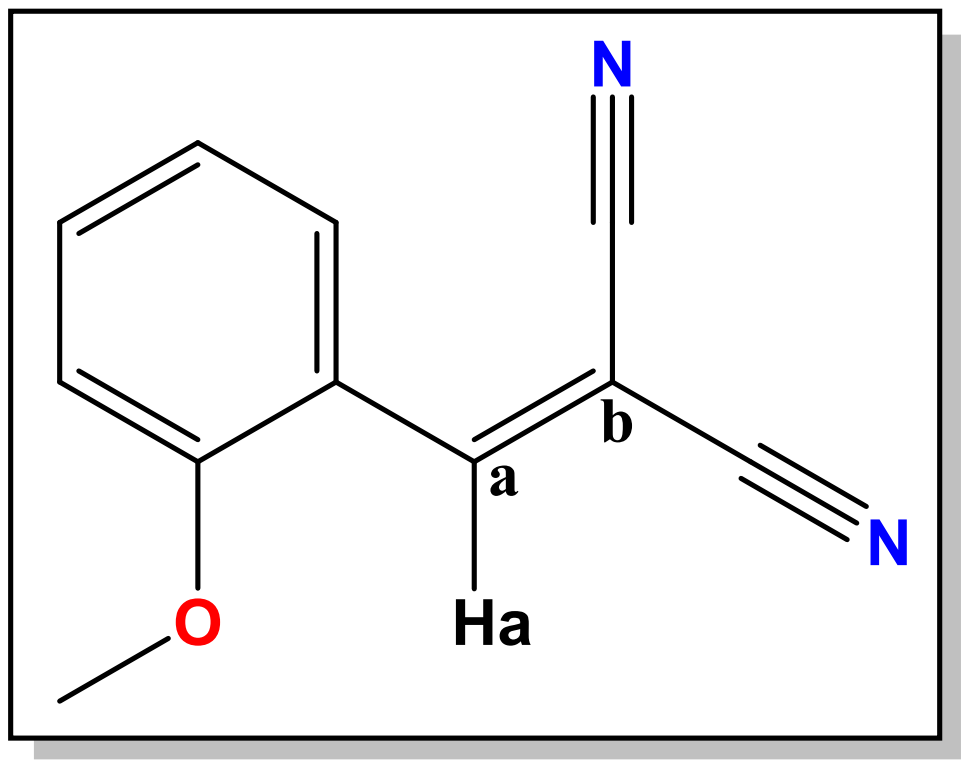

Pale yellow solid; **Yield** = 80%; **m.p.**: 135-137 °C.

^{1}H NMR (300 MHz, DMSO-d$_6$) (δ in ppm): 3.91 (s, 3H, CH_3), 7.15 (t, 1H, *J* = 7.89 Hz, Ar–H), 7.24 (d, 1H, *J* = 7.98 Hz, Ar–H), 7.69 (td, 1H, *J* = 1.65 Hz, *J* = 7.32 Hz, Ar–H), 7.97 (dd, 1H, *J* = 1.68 Hz, *J* = 7.32 Hz, Ar–H), 8.48 (s, 1H, Ha),

^{13}C NMR (75 MHz, DMSO-d$_6$) (δ in ppm): 56.58 (CH_3), 82.06 (C_b), 112.93, 113.73, 114.88, 120.39, 121.40, 156.55 (C-CH_3), 159.12 (C_a).

***2-(4-chlorobenzylidene)malononitrile* 3:**

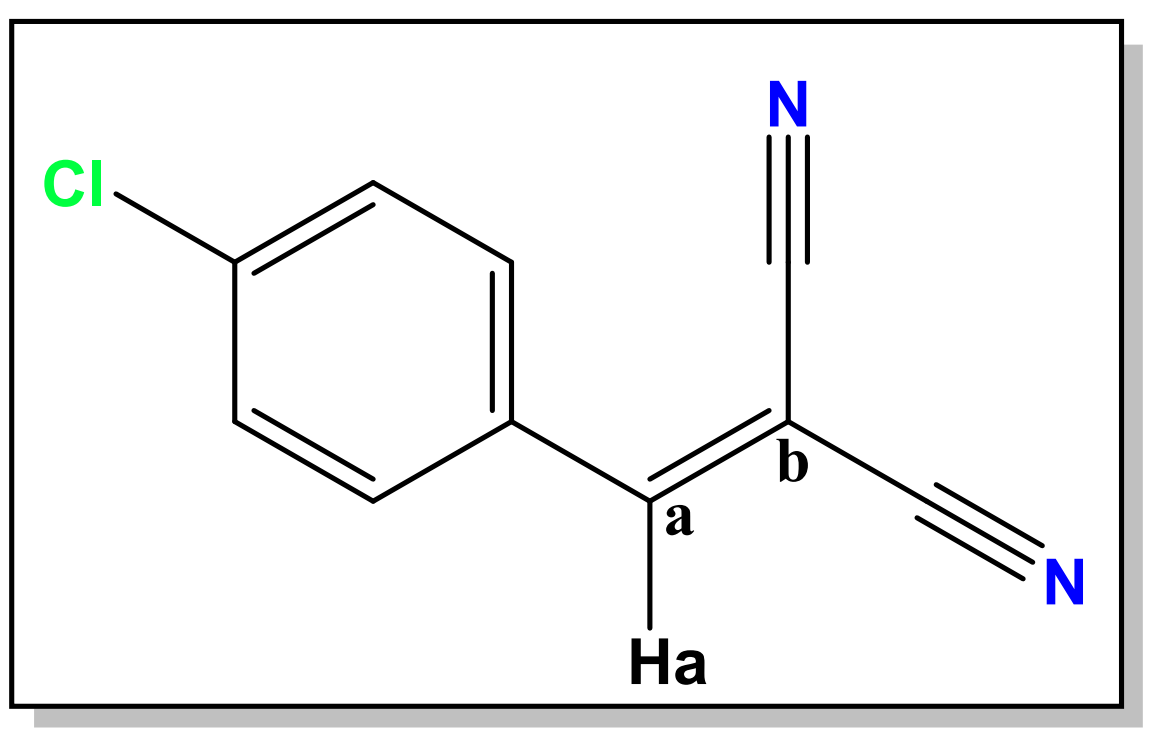

White solid; **Yield** = 96%; **m.p.**: 168-170 °C.

^{1}H NMR (300 MHz, DMSO-d$_6$) (δ in ppm): 7.72 (d, 2H, *J* = 8.64 Hz, Ar–H), 7.97 (d, 1H, *J* = 8.94 Hz, Ar–H), 8.55 (s, 1H, Ha),

^{13}C NMR (75 MHz, DMSO-d$_6$) (δ in ppm): 82.76 (C_b), 113.48, 114.52, 130.20, 130.57, 132.60, 139.47 (C-Cl), 160.59 (C_a).

2-(3-nitrobenzylidene)malononitrile **4**:

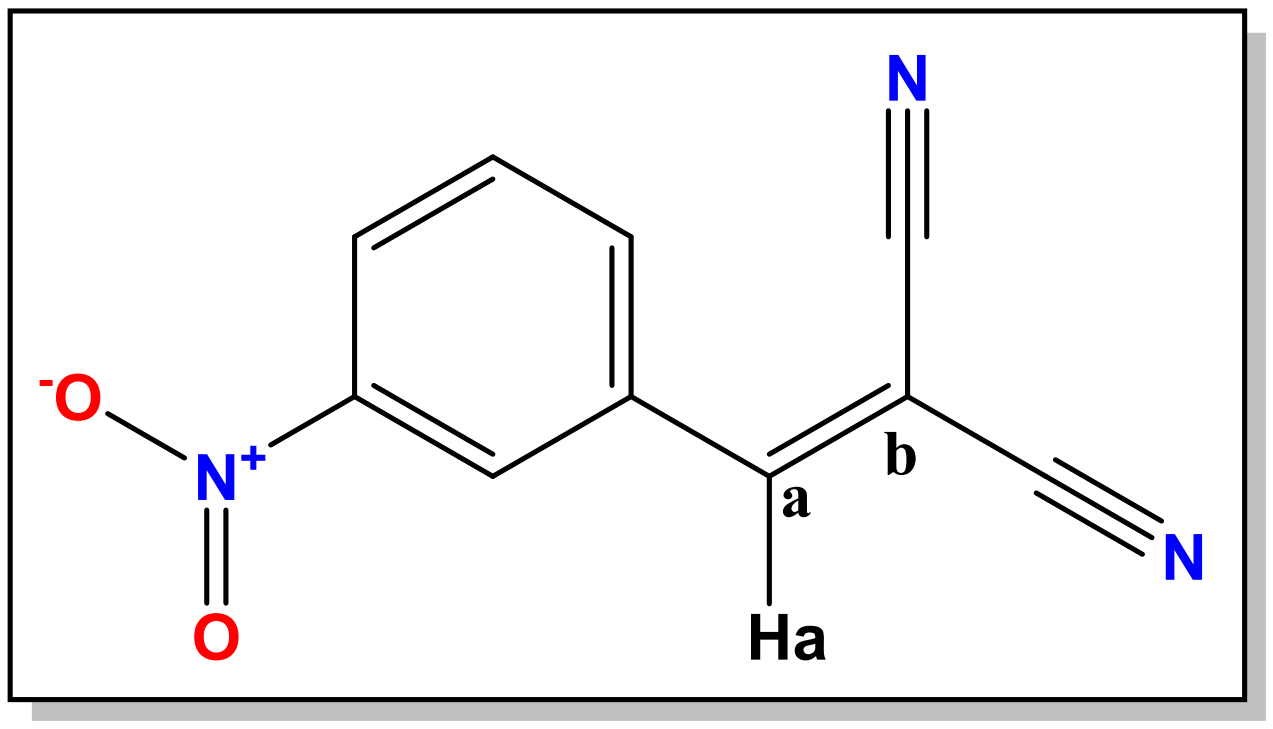

Pale yellow solid; **Yield** = 75%; **m.p.**: 108-110 °C.

^{1}H NMR (300 MHz, DMSO-d_6) (δ in ppm): 7.93 (t, 1H, *J* = 8.04 Hz, Ar–H), 8.33 (dt, 1H, *J* = 0.84 Hz, *J* = 1.77 Hz, Ar–H), 8.49 (dd, 1H, *J* = 1.02 Hz, *J* = 2.32 Hz, Ar–H), 8.73 (s, 1H, Ha), 8.78 (t, 1H, *J* = 1.77 Hz, Ar–H).

^{13}C NMR (75 MHz, DMSO-d_6) (δ in ppm): 85.42 (C_b), 113.08, 114.14, 125.30, 128.39, 131.64, 132.93, 136.33, 148.51 (C-NO_2), 159.77 (C_a).

2-(4-bromobenzylidene)malononitrile **5:**

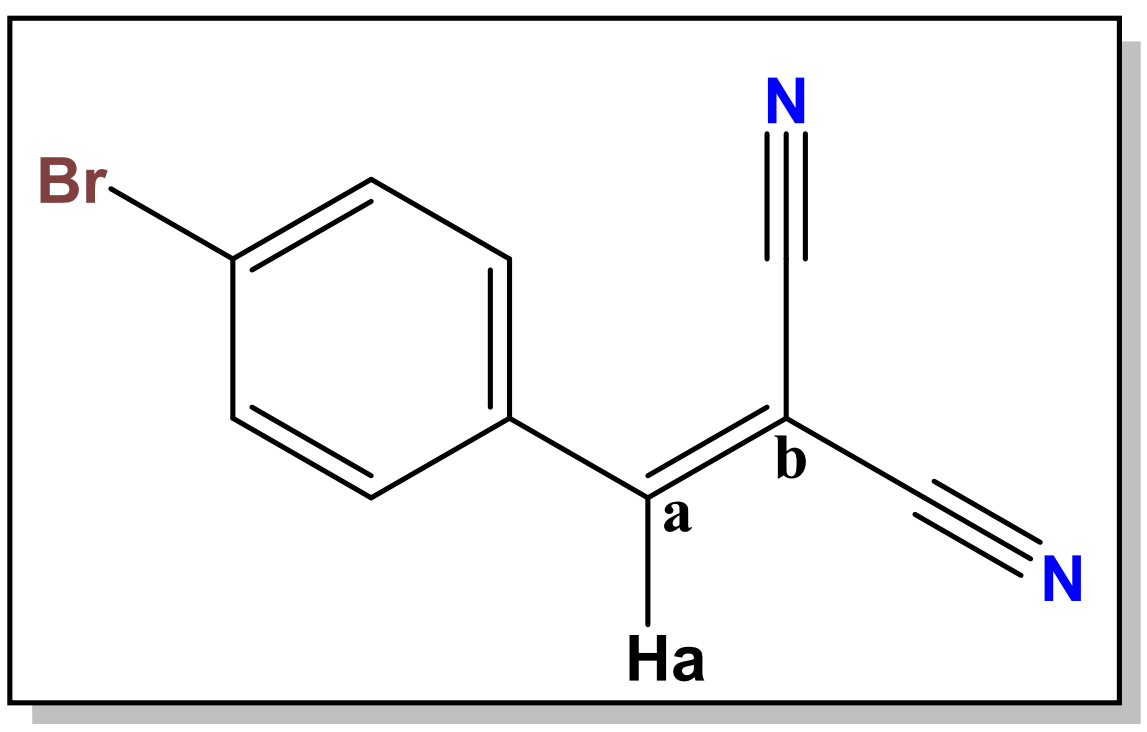

White solid; **Yield** = 87%; **m.p.**: 156-158 °C.

^{1}H NMR (300 MHz, DMSO-d_6) (δ in ppm): 7.87 (s, 4H, Ar–H), 8.54 (s, 1H, Ha).

^{13}C NMR (75 MHz, DMSO-d_6) (δ in ppm): 85.80 (C_b), 113.49, 114.55, 128.77, 130.86, 132.59, 133.15, 160.76 (C_a).

2-(4-(dimethylamino)benzylidene)malononitrile **6:**

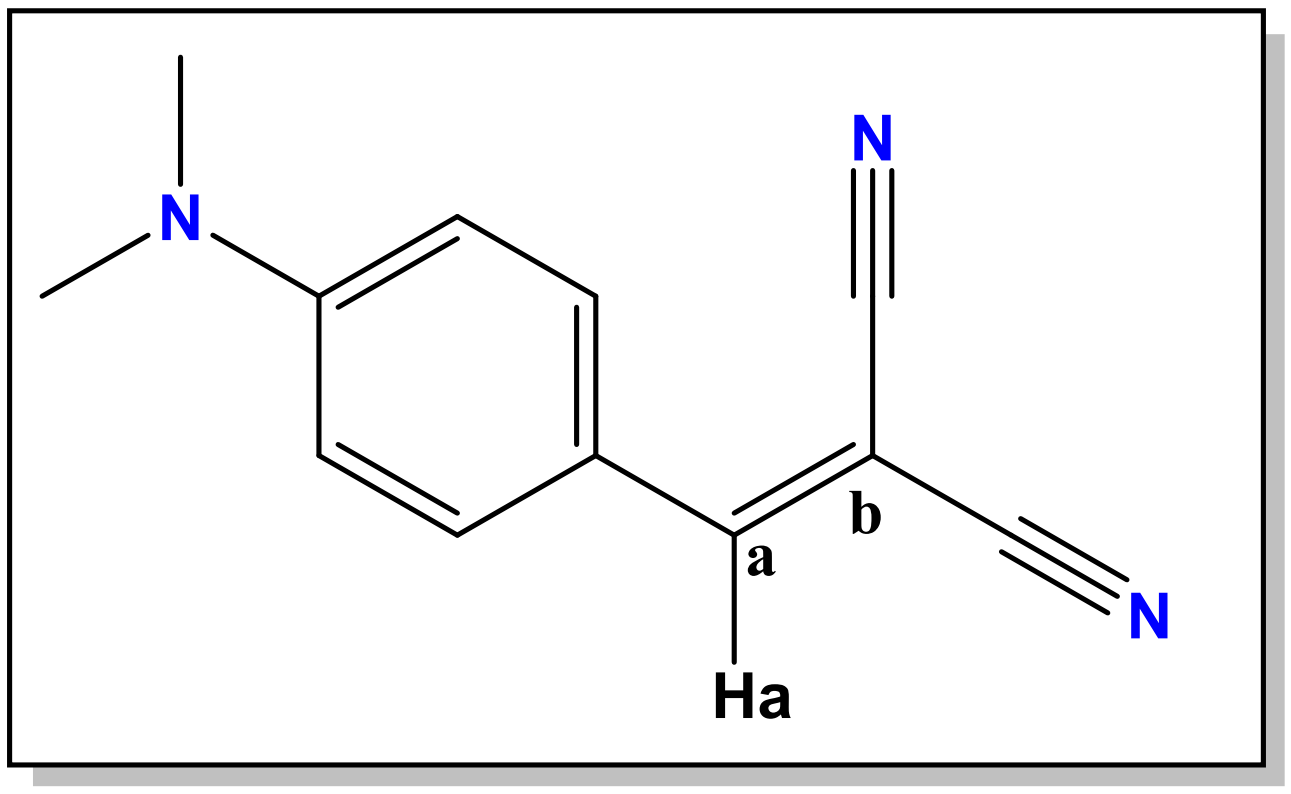

Orange-yellow solid; **Yield** = 75%; **m.p.**: 170-172 °C.

^{1}H NMR (300 MHz, DMSO-d$_6$) (δ in ppm): 3.11 (s, 6H, CH_3), 6.79 (dt, 1H, J = 2.88 Hz, J = 4.89 Hz, Ar–H), 6.87 (dt, 1H, J = 3.21 Hz, J = 5.19 Hz, Ar–H), 7.69 (dt, 1H, J = 2.76 Hz, J = 4.83 Hz, Ar–H), 7.85 (dt, 1H, J = 3.27 Hz, J = 4.83 Hz, Ar–H), 8.06 (s, 1H, Ha).

^{13}C NMR (75 MHz, DMSO-d$_6$) (δ in ppm): 87.62 (C_b), 99.99, 111.54, 112.29, 119.21, 134.07, 146.23, 154.82 (C-N(CH_3)$_2$), 159.35 (C_a).

***2-(2-nitrobenzylidene)malononitrile* 7:**

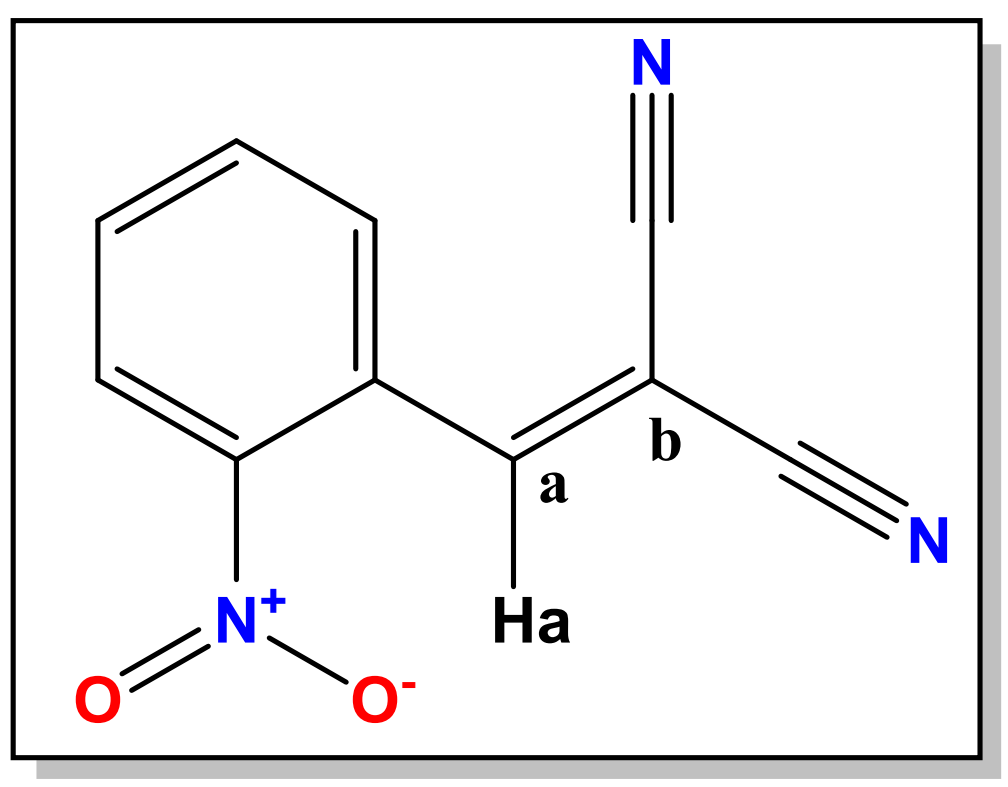

Yellow solid; **Yield** = 72%; **m.p.**: 142-144 °C.

^{1}H NMR (300 MHz, DMSO-d$_6$) (δ in ppm): 7.90-8.02 (m, 3H, Ar–H), 6.35 (dd, 1H, J = 0.96 Hz, J = 8.34 Hz, Ar–H), 8.98 (s, 1H, Ha).

^{13}C NMR (75 MHz, DMSO-d$_6$) (δ in ppm): 80.62 (C_b), 113.58, 125.91, 127.63, 130.92, 133.84, 135.53, 147.67 (C-N(CH_3)$_2$), 161.82 (C_a).

***2-(4-hydroxy-3-methoxybenzylidene)malononitrile* 8:**

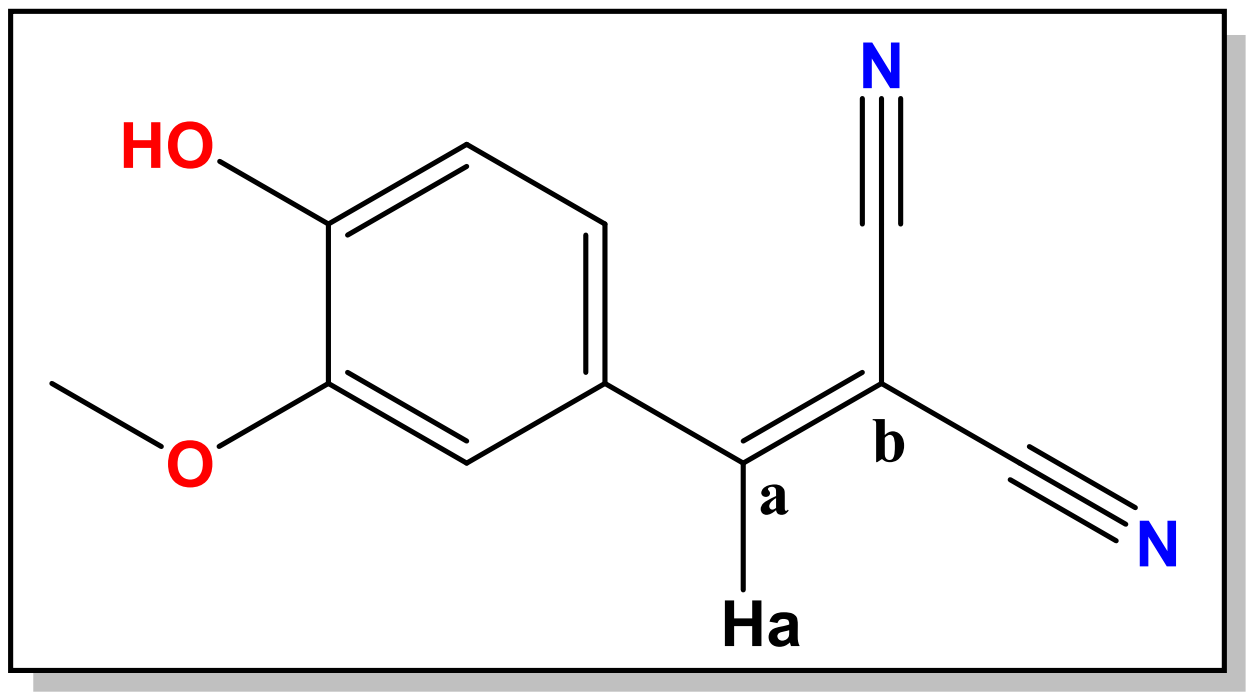

Yellowish-brown solid; **Yield** = 69%; **m.p.**: 127-129 °C.

^{1}H NMR (300 MHz, DMSO-d$_6$) (δ in ppm): 3.81 (s, 3H, OCH_3), 6.99 (d, 1H, J = 8.34 Hz, Ar–H), 7.51 (dd, 1H, J = 2.10 Hz, J = 8.49 Hz, Ar–H), 7.64 (d, 1H, J = 2.13 Hz, Ar–H), 8.2 (s, 1H, Ha), 10.70 (s, 1H, OH).

^{13}C NMR (75 MHz, DMSO-d$_6$) (δ in ppm): 56.02 (OCH_3), 75.37 (C_b), 113.60, 114.82, 115.58, 116.63, 123.49, 128.15, 148.37 (C-OH), 154.41 (C-OCH_3), 161.04 (C_a).

2.2 Copies of the (^{1}H, ^{13}C) NMR spectra of all compounds (1-8)

***2-(4-hydroxybenzylidene)malononitrile* 1:**

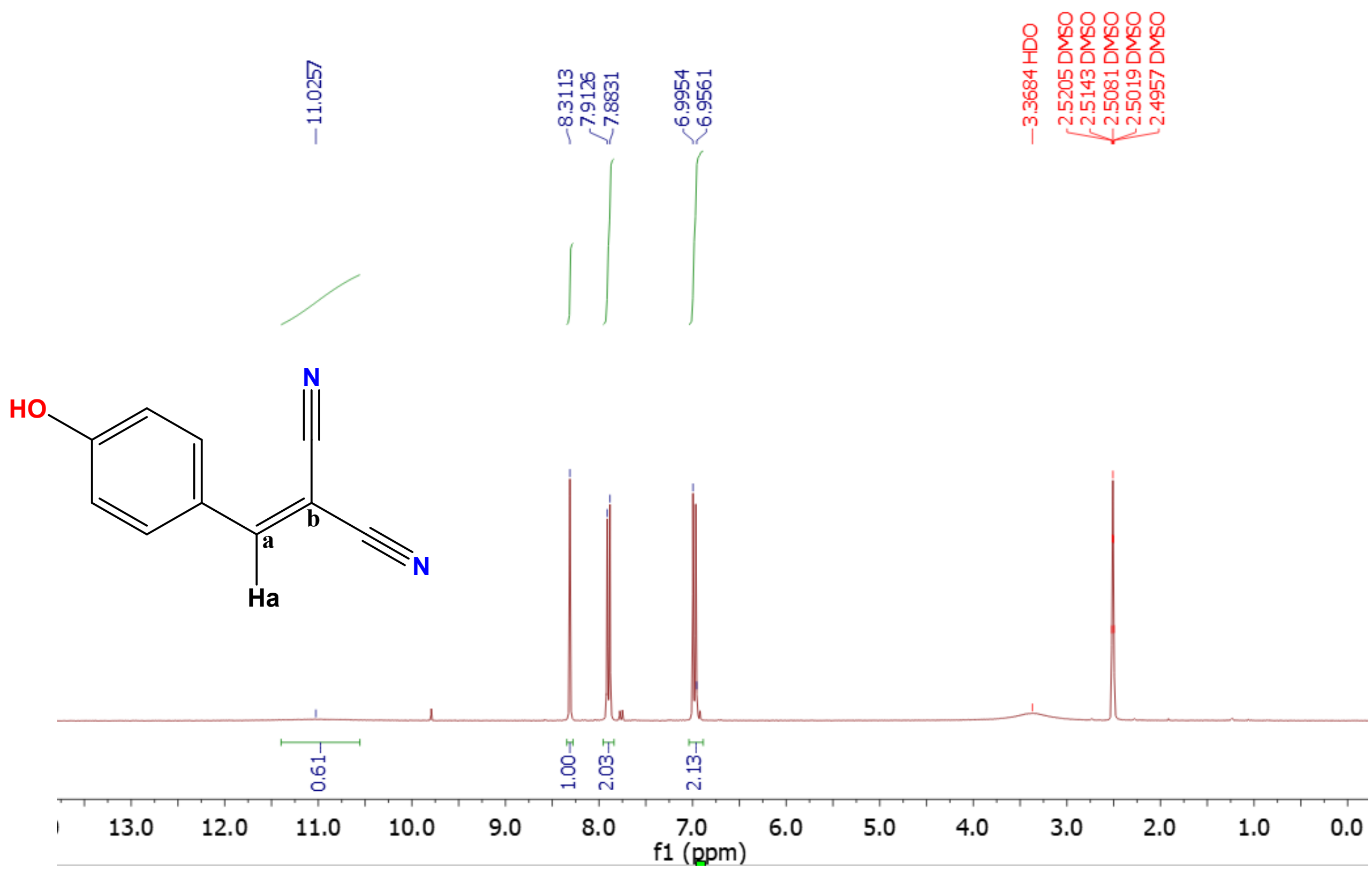

Figure S1. ^{1}H NMR spectrum (300 MHz, **DMSO-d$_6$**) of compound **1**

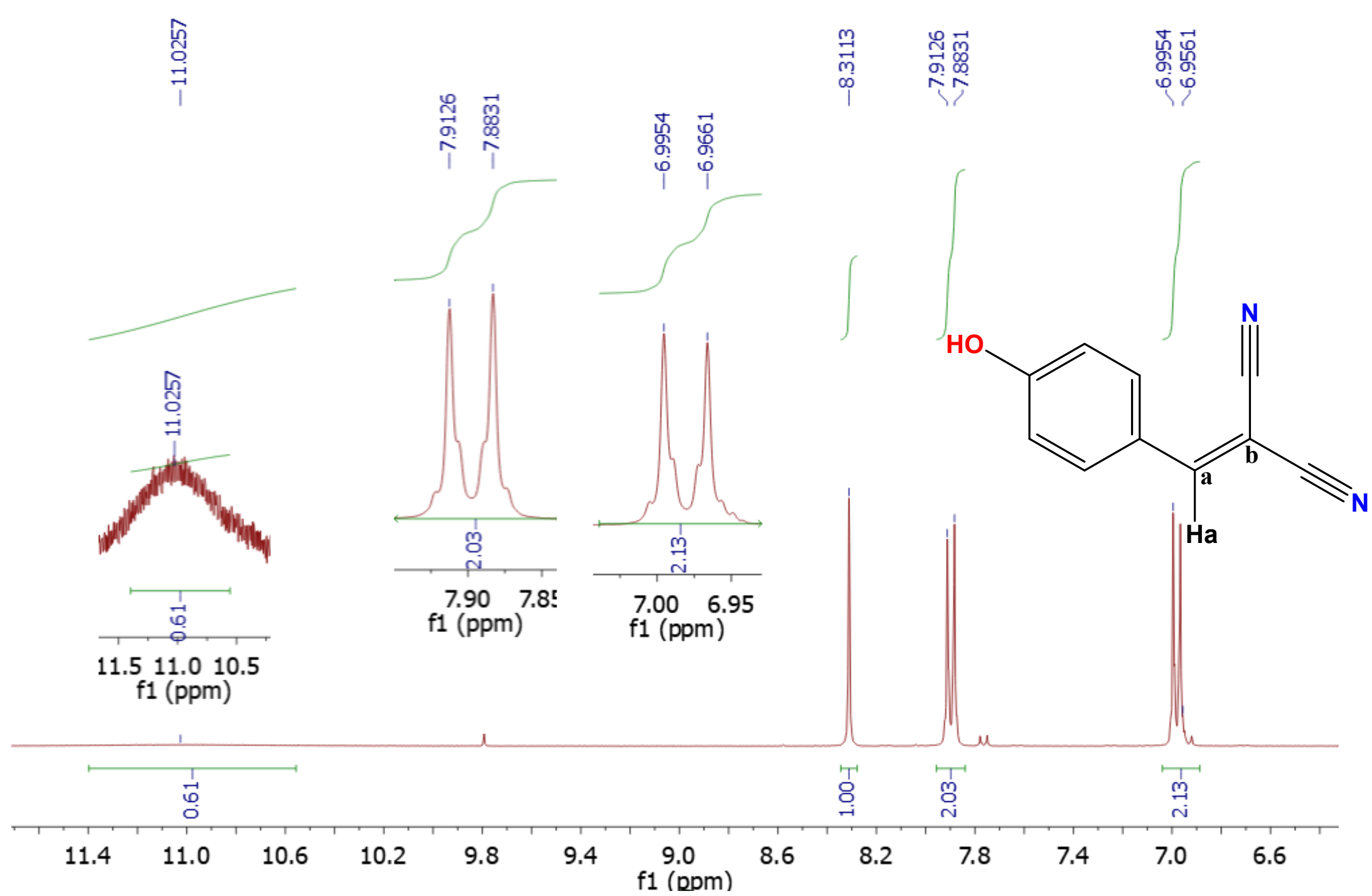

Figure S2. Aromatic enlarged region of ^{1}H NMR spectrum of compound **1**

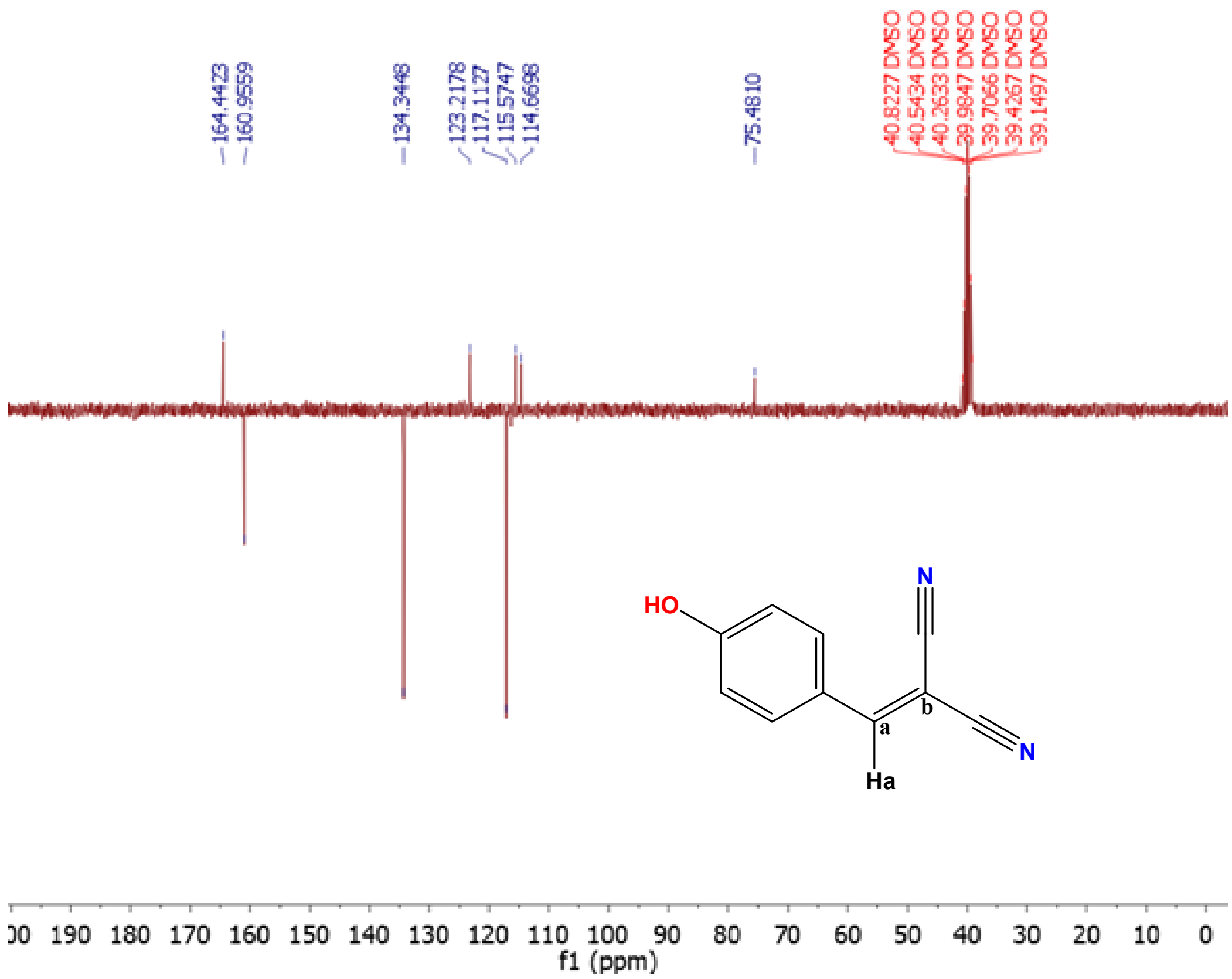

Figure S3. ^{13}C NMR spectrum (75 MHz, **DMSO-d$_6$**) of compound **1**

***2-(2-methoxybenzylidene)malononitrile* 2:**

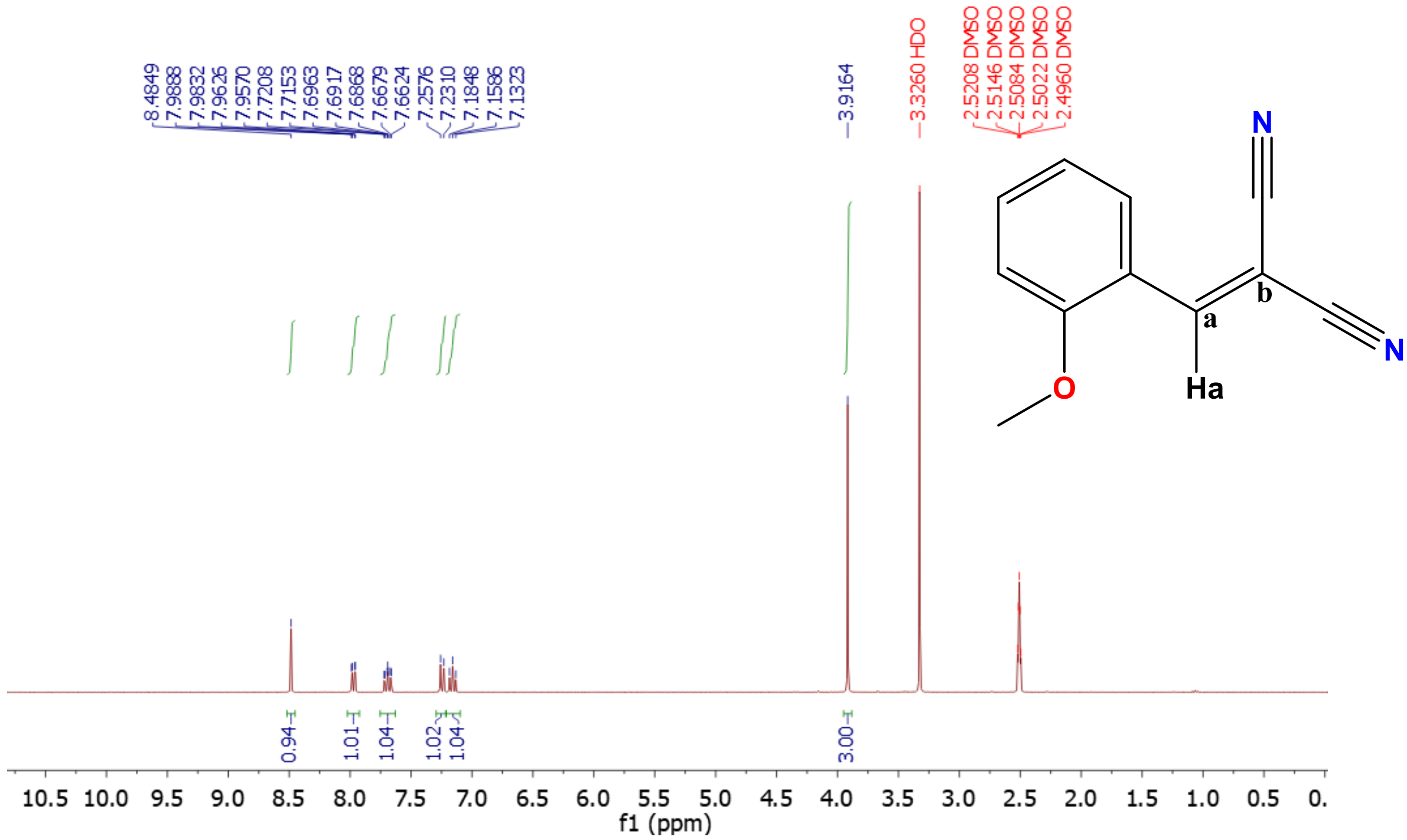

Figure S4. ^{1}H NMR spectrum (300 MHz, **DMSO-d$_6$**) of compound **2**

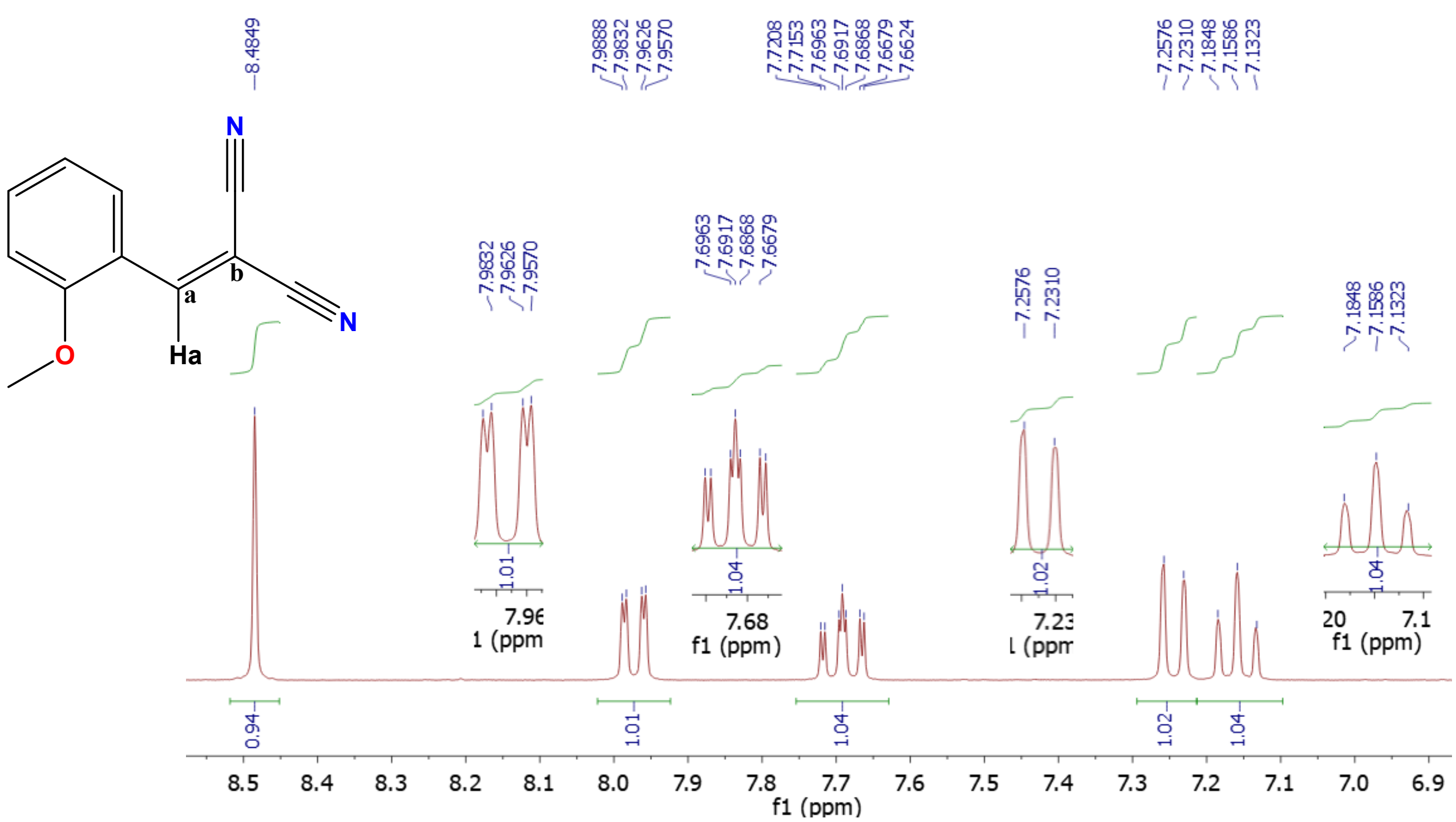

Figure S5. Aromatic enlarged region of ^{1}H NMR spectrum of compound **2**

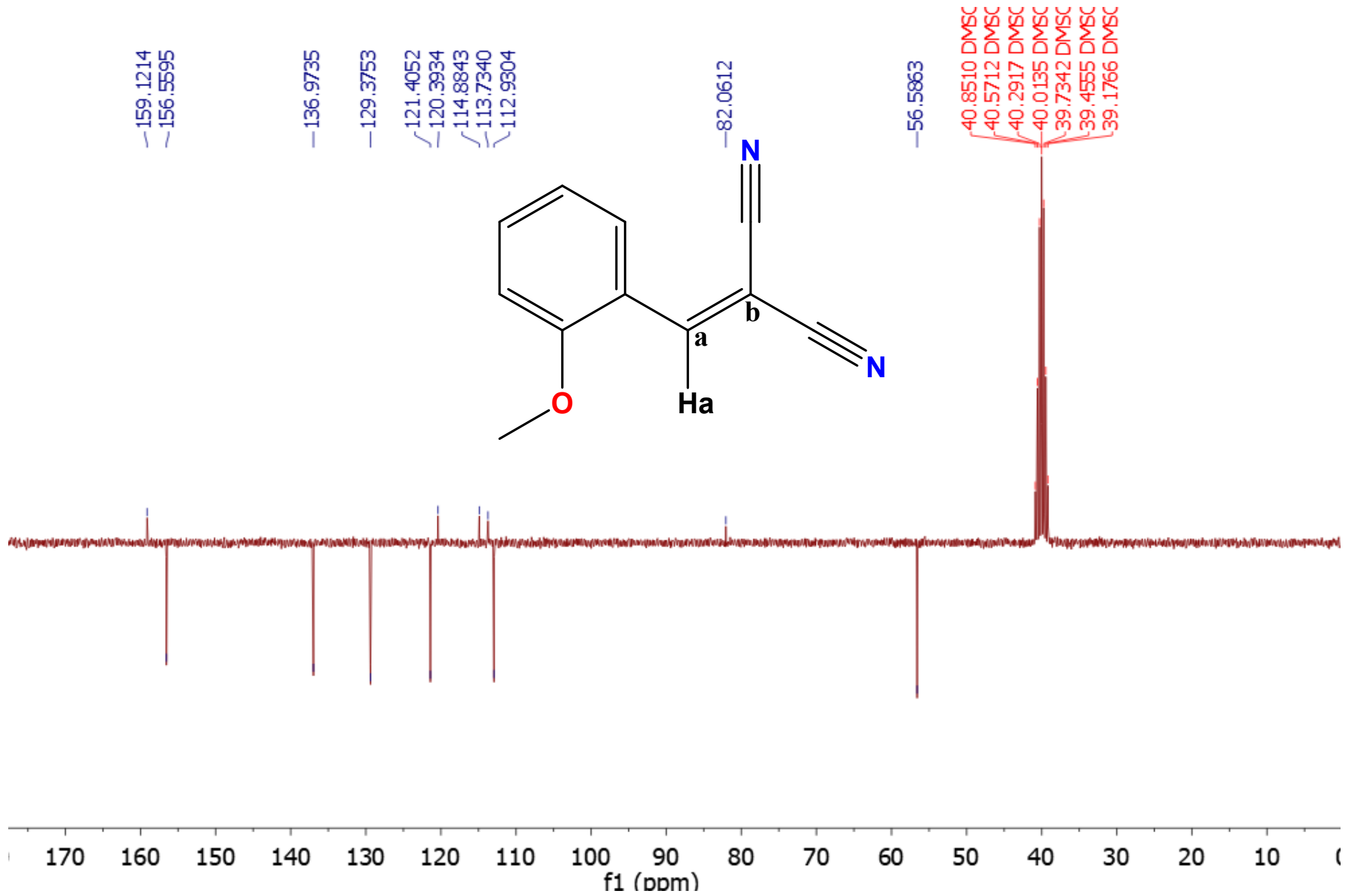

Figure S6. ^{13}C NMR spectrum (75 MHz, **DMSO-d$_6$**) of compound **2**

4.1 ***2-(4-chlorobenzylidene)malononitrile*** **3:**

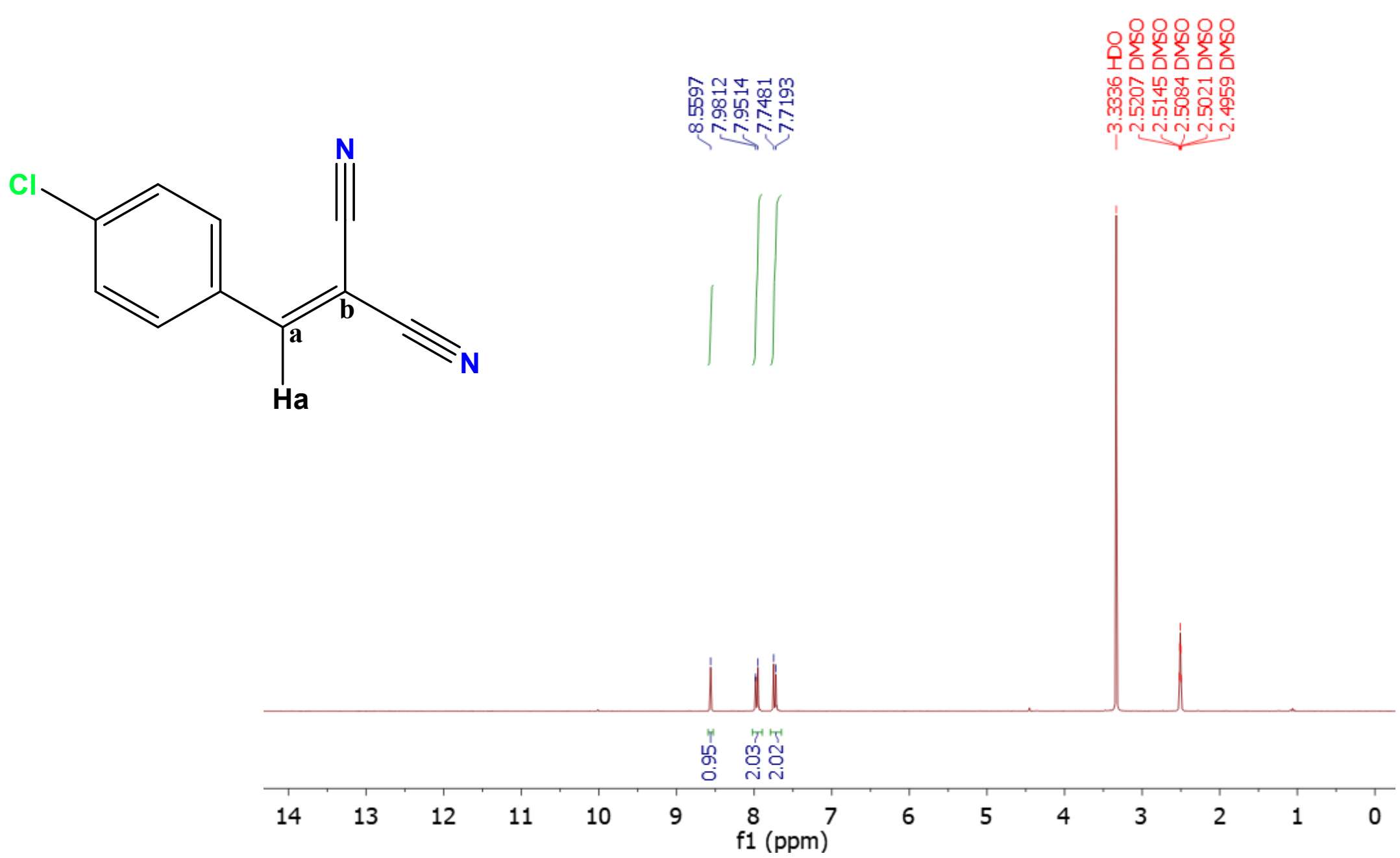

Figure S7. ^{1}H NMR spectrum (300 MHz, **DMSO-d$_6$**) of compound **3**

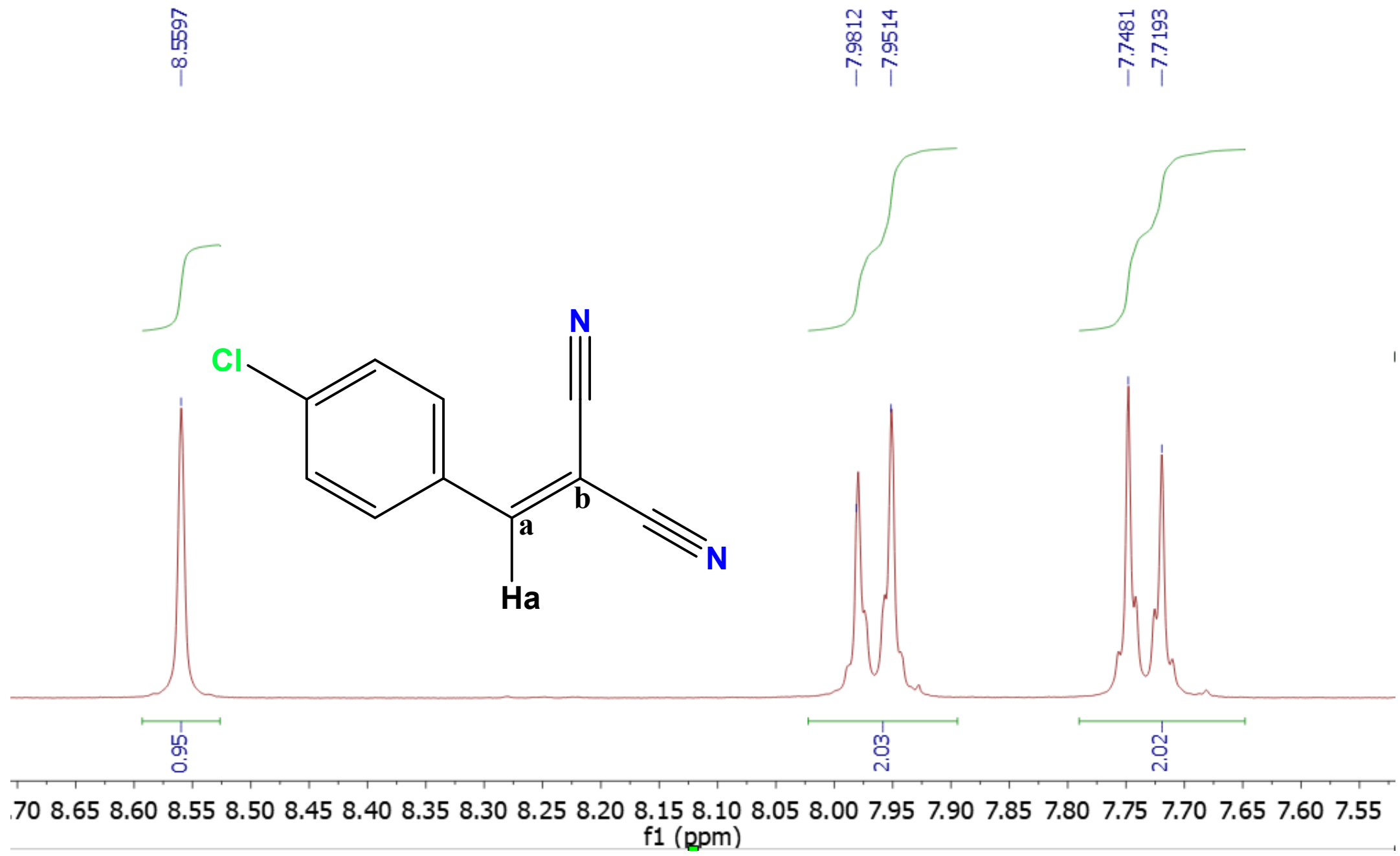

Figure S8. Aromatic enlarged region of ^{1}H NMR spectrum of compound **3**

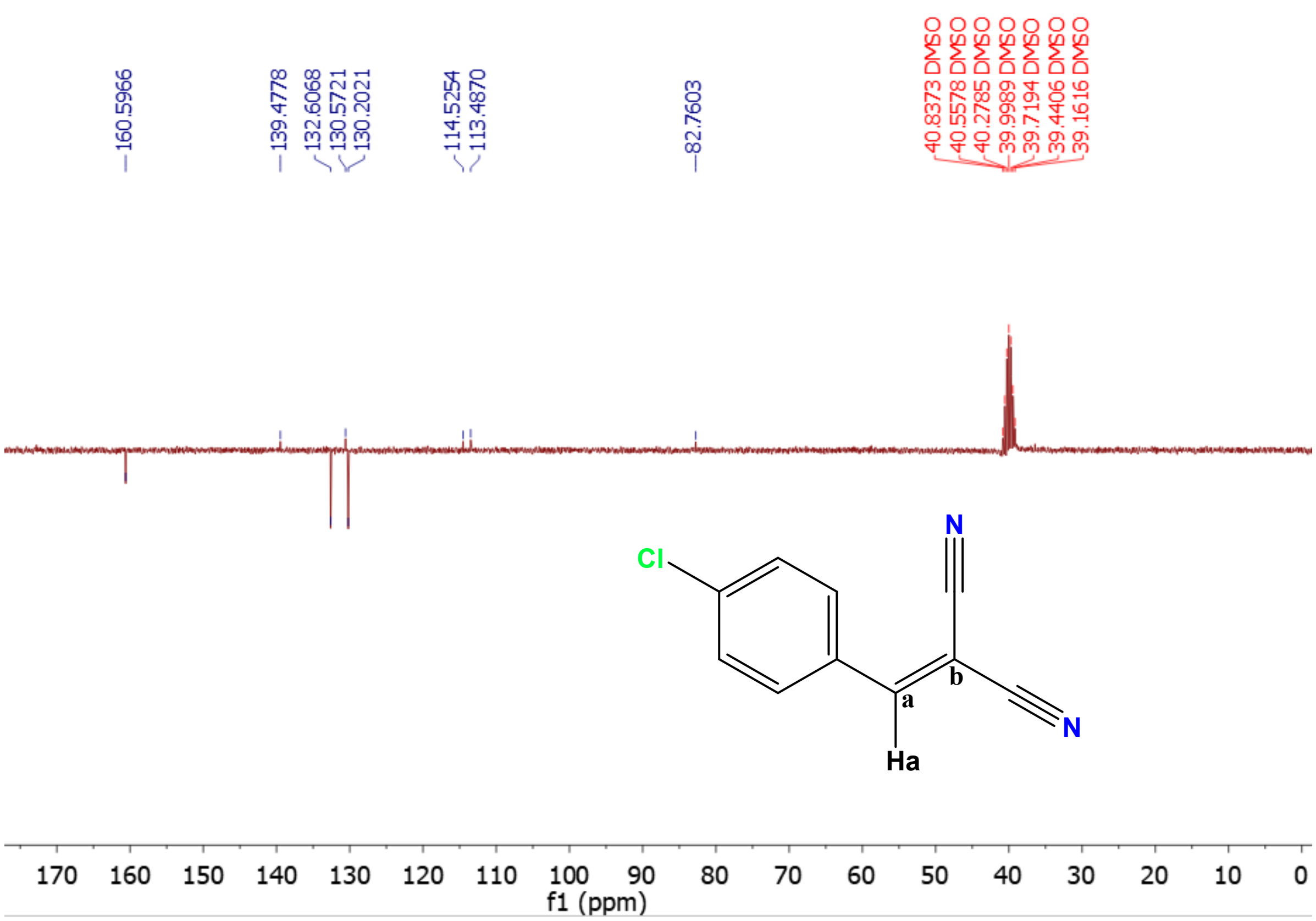

Figure S9. ^{13}C NMR spectrum (75 MHz, **DMSO-d$_6$**) of compound **3**

4.2 ***2-(3-nitrobenzylidene)malononitrile* 4:**

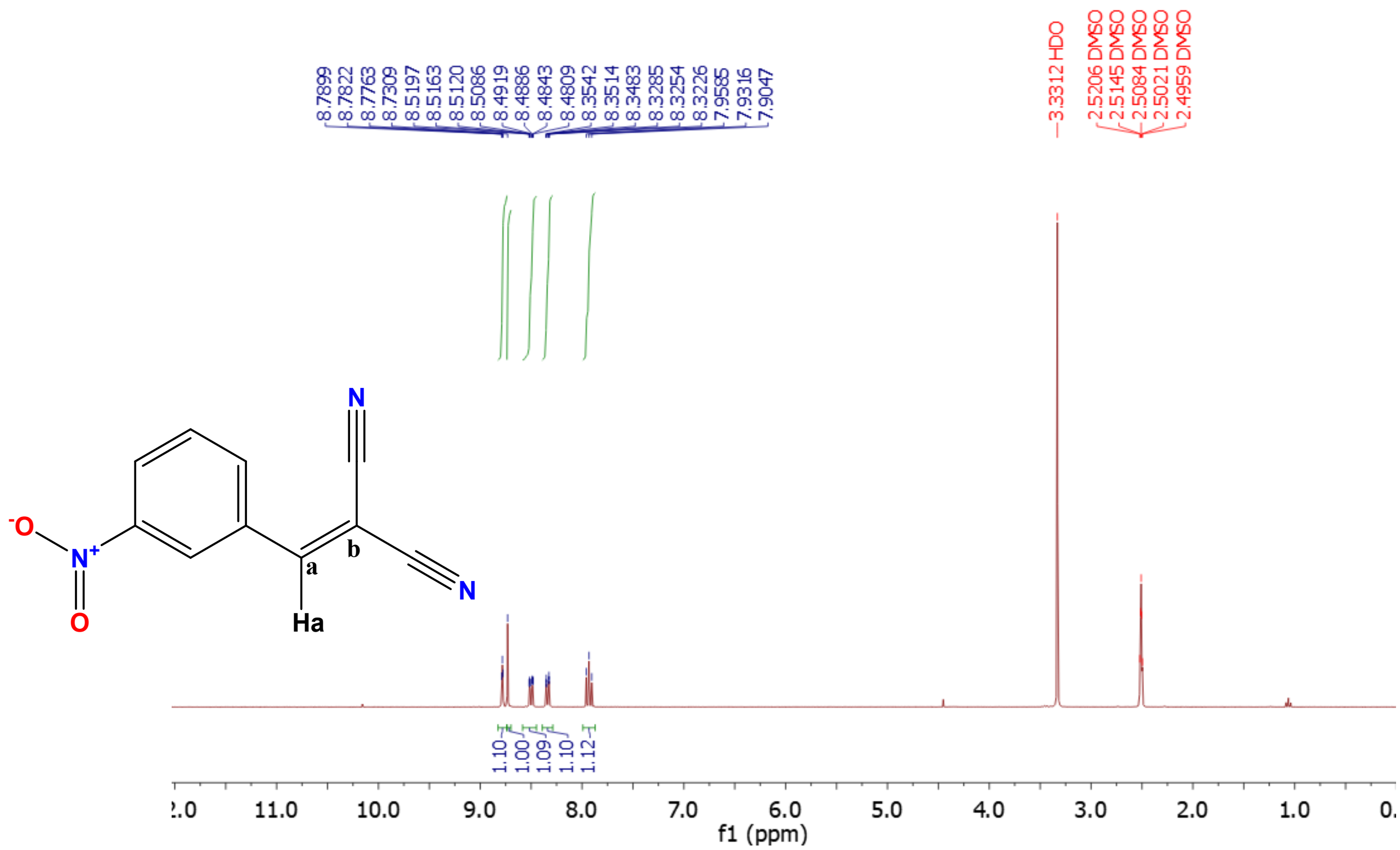

Figure S10. ^{1}H NMR spectrum (300 MHz, **DMSO-d$_6$**) of compound **4**

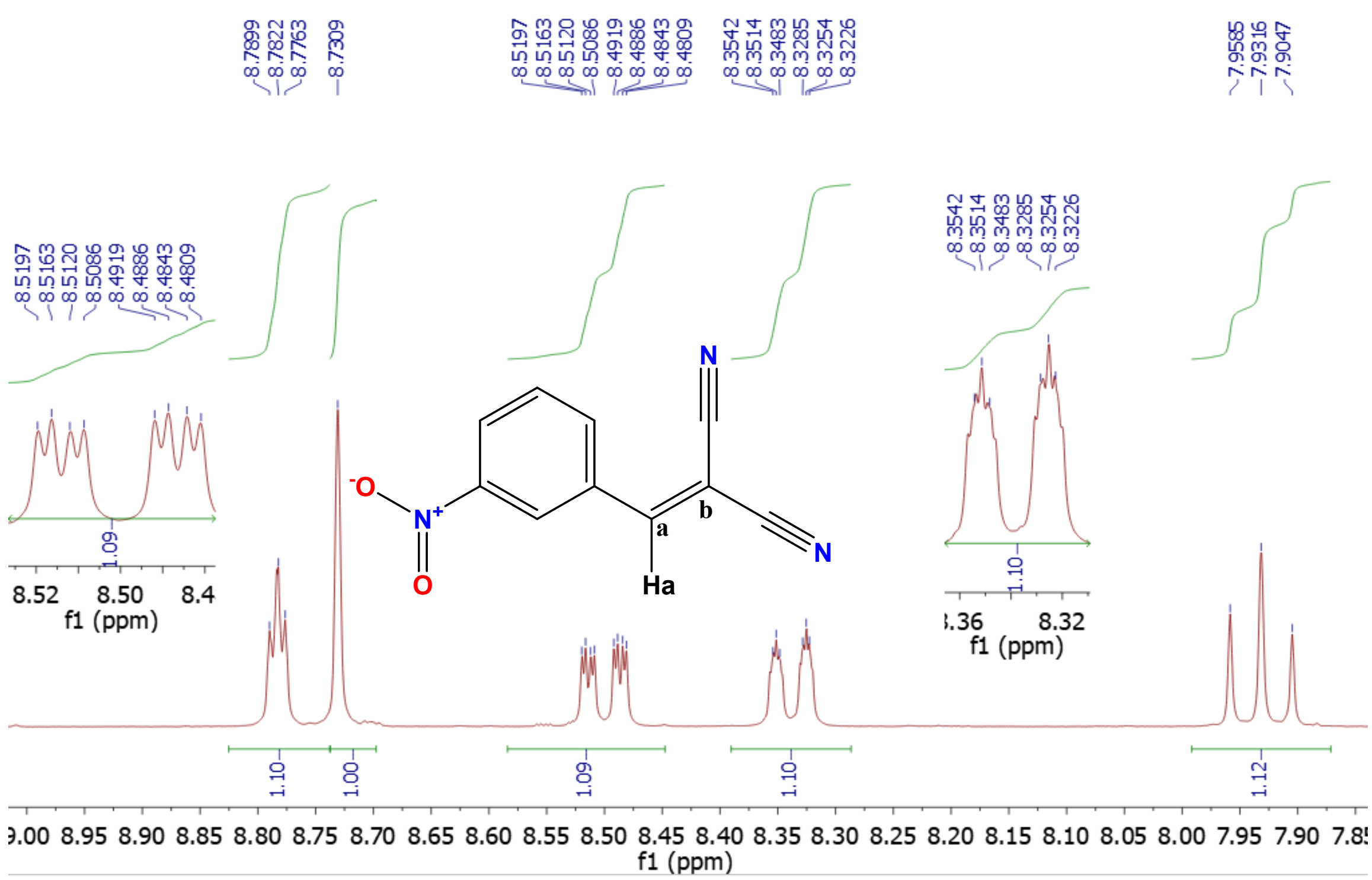

Figure S11. Aromatic enlarged region of ^{1}H NMR spectrum of compound **4**

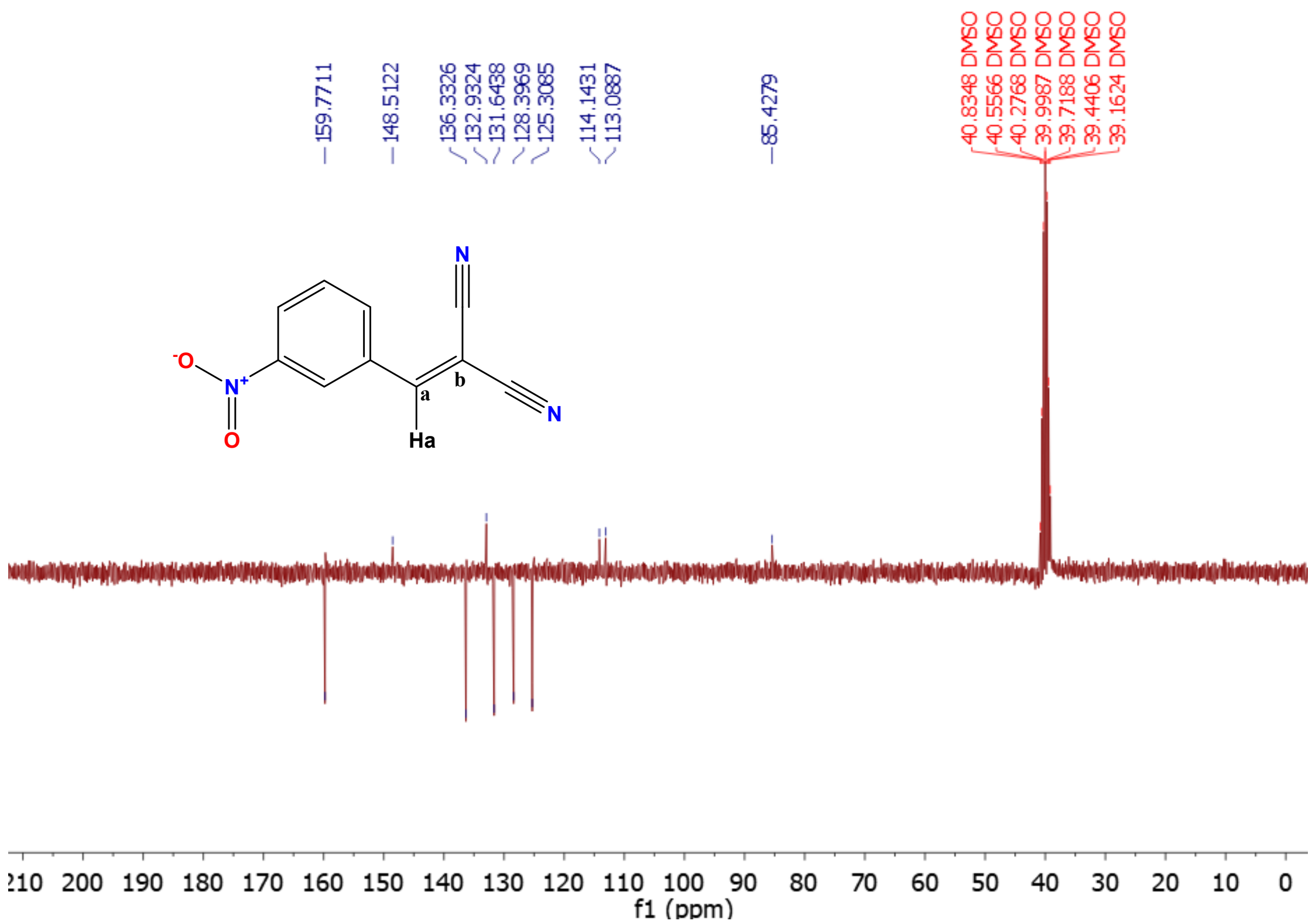

Figure S12. ^{13}C NMR spectrum (75 MHz, **DMSO-d$_6$**) of compound **4**

4.3 ***2-(4-bromobenzylidene)malononitrile*** **5:**

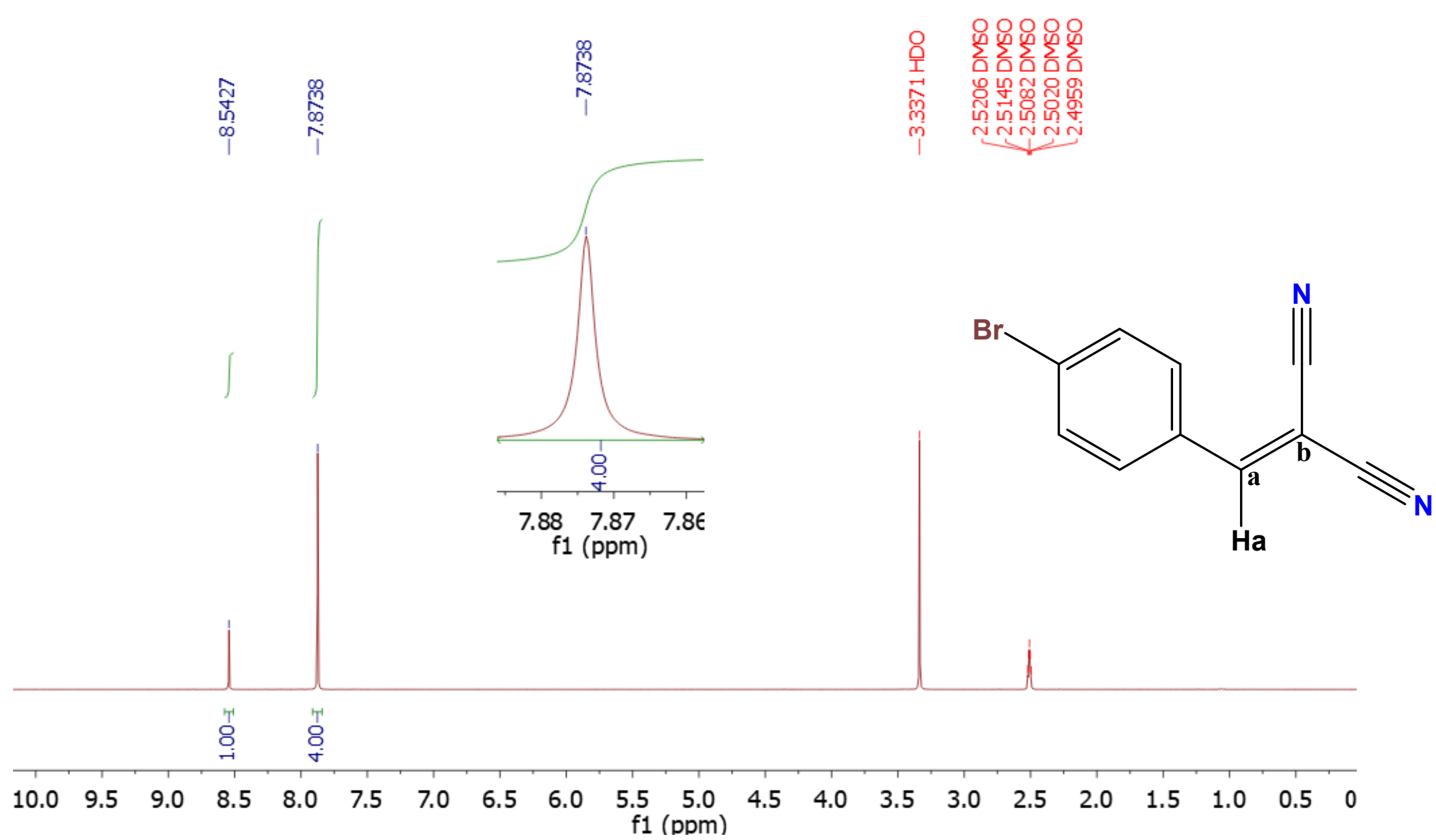

Figure S13. ^{1}H NMR spectrum (300 MHz, **DMSO-d$_6$**) of compound **5**

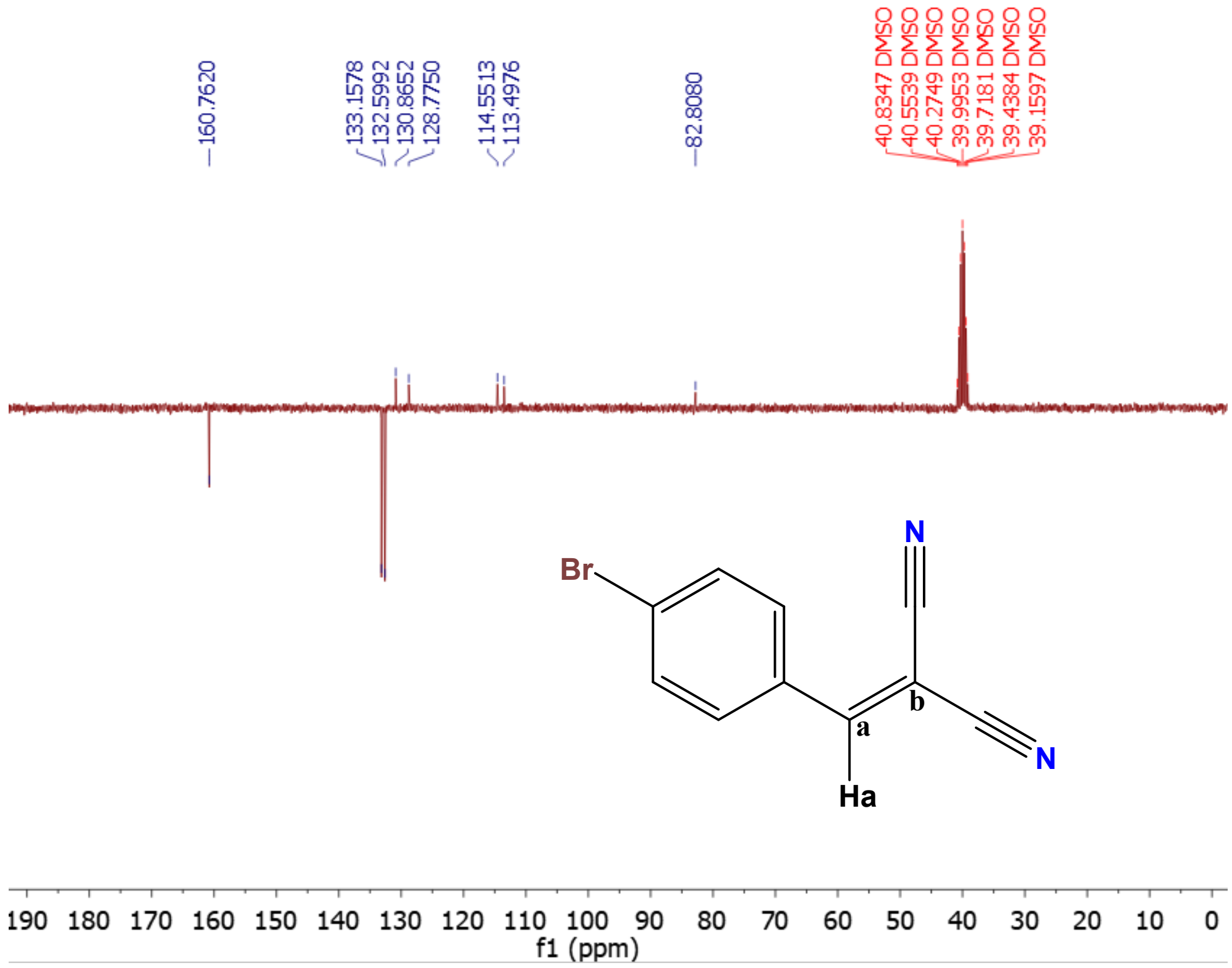

Figure S14. ^{13}C NMR spectrum (75 MHz, **DMSO-d$_6$**) of compound **5**

***2-(4-(dimethylamino)benzylidene)malononitrile* 6:**

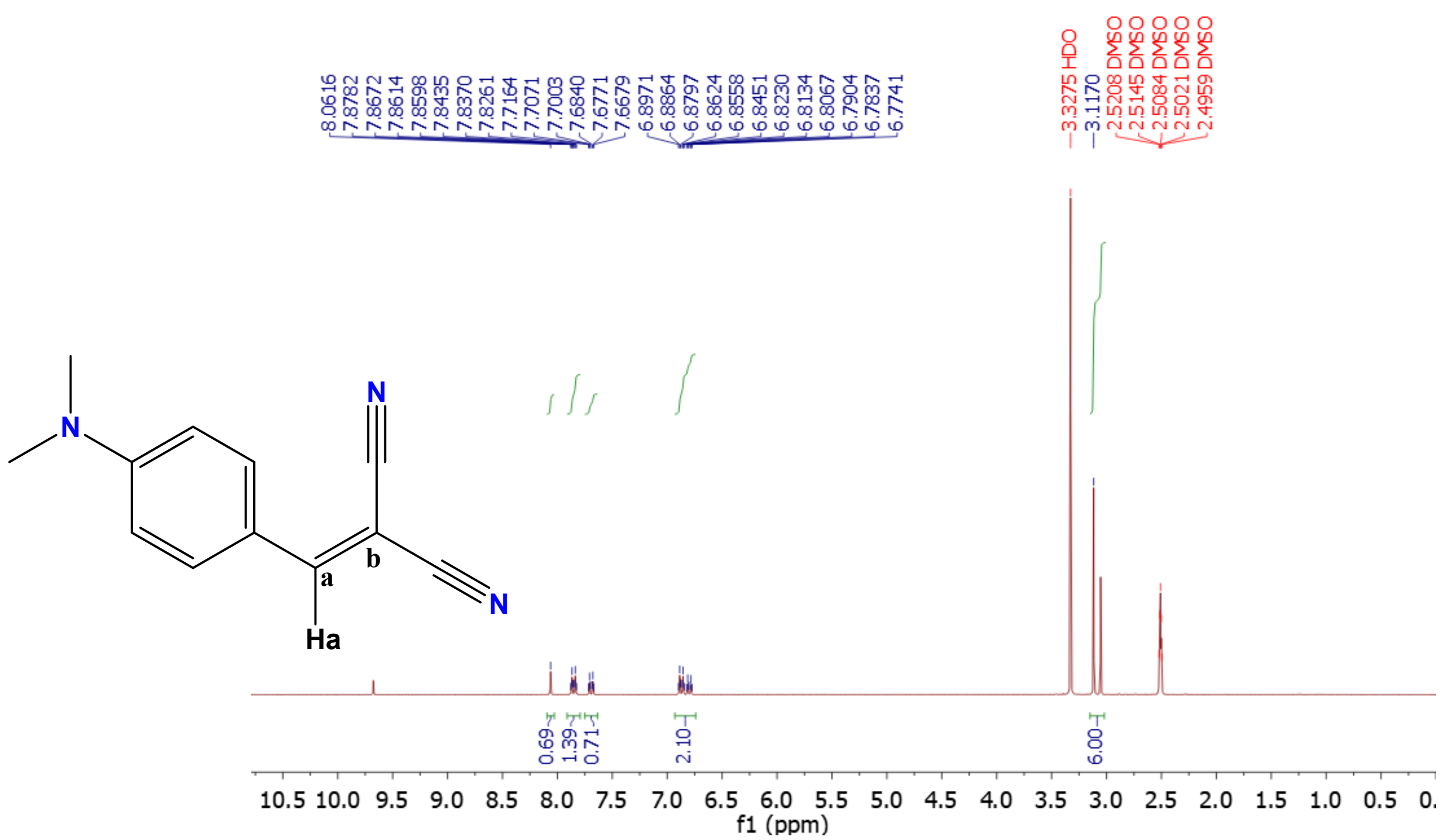

Figure S15. ^{1}H NMR spectrum (300 MHz, **DMSO-d$_6$**) of compound **6**

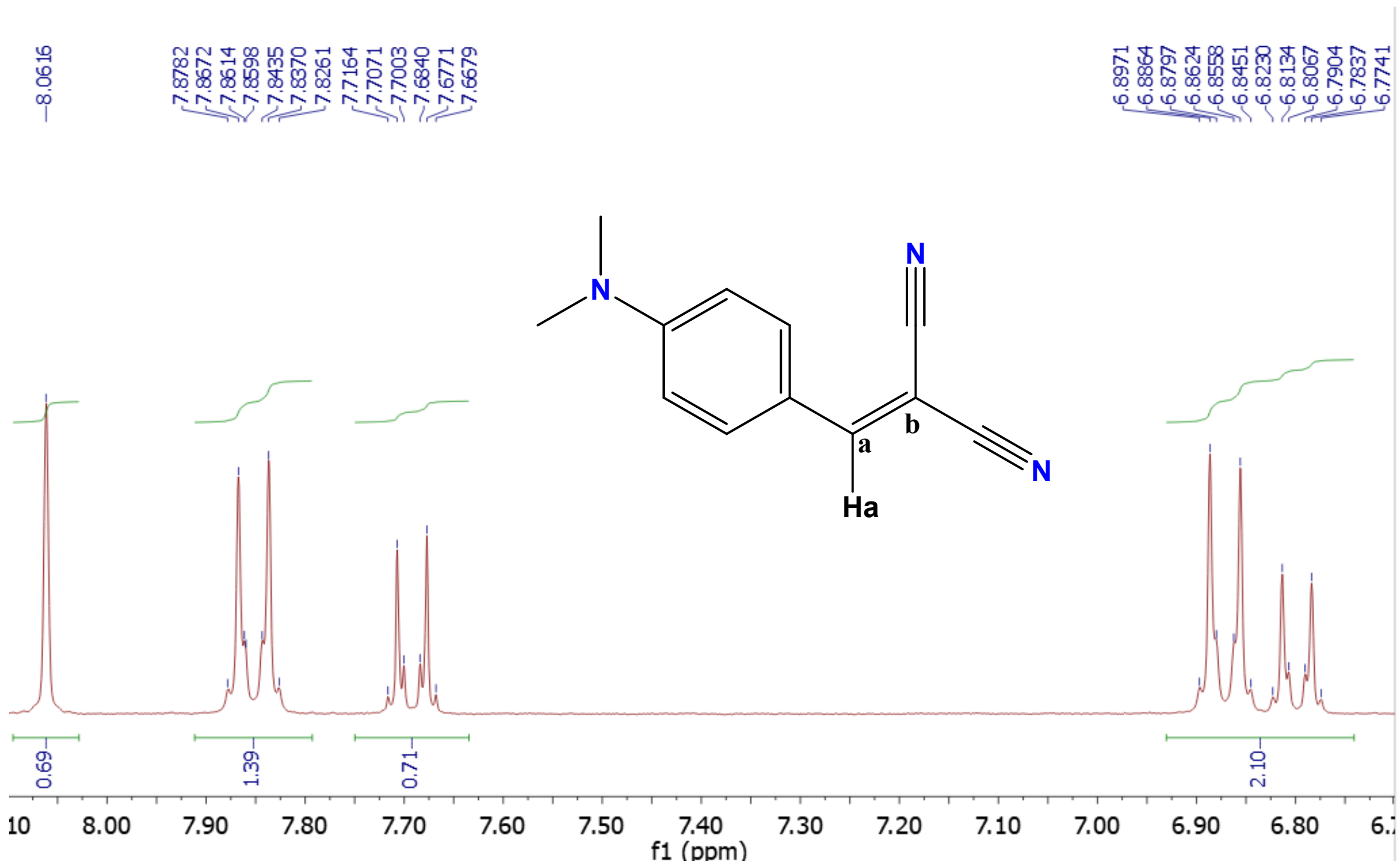

Figure S16. Aromatic enlarged region of ^{1}H NMR spectrum of compound **6**

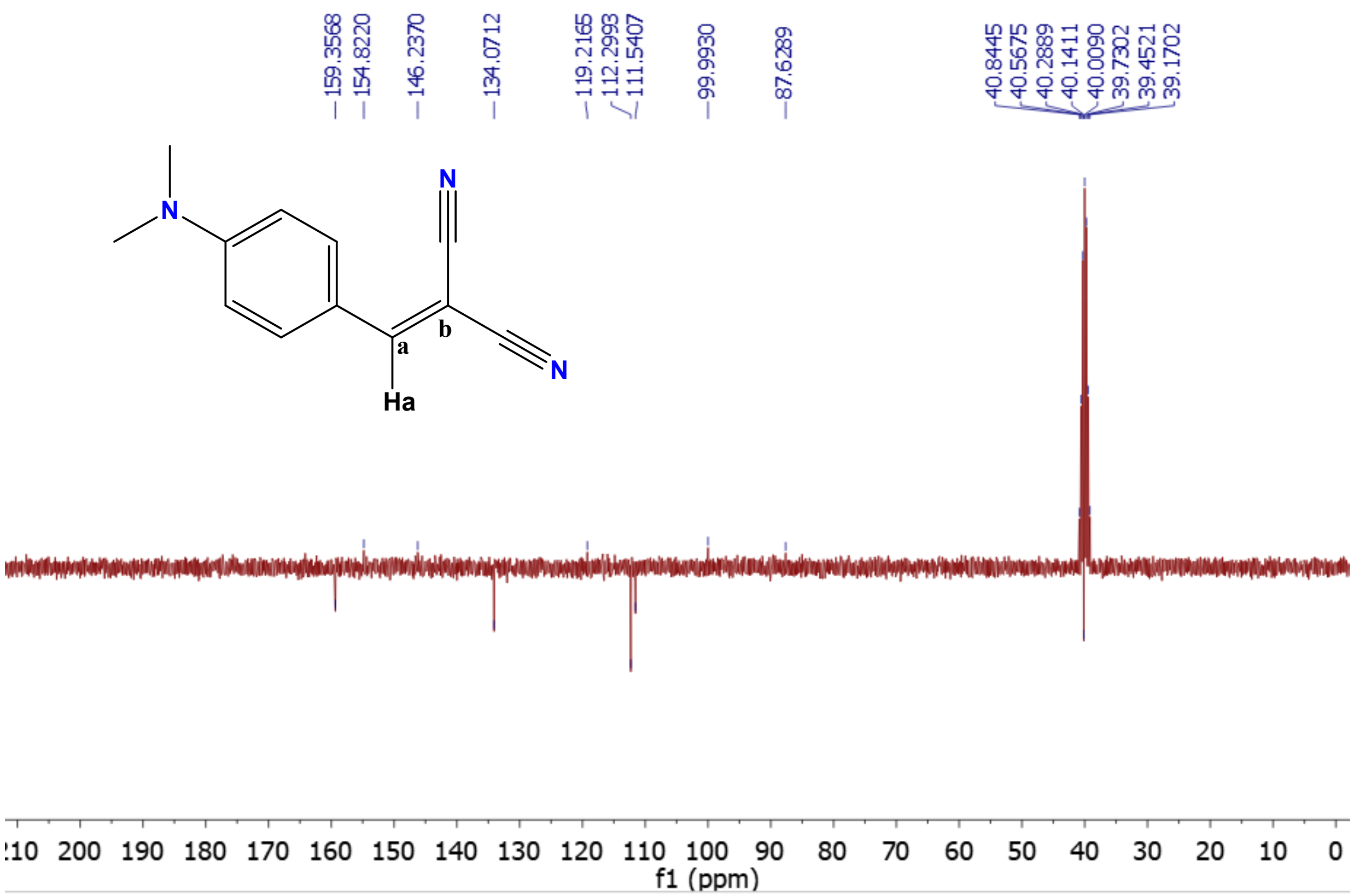

Figure S17. ^{13}C NMR spectrum (75 MHz, **DMSO-d$_6$**) of compound **6**

4.4 ***2-(2-nitrobenzylidene)malononitrile* 7:**

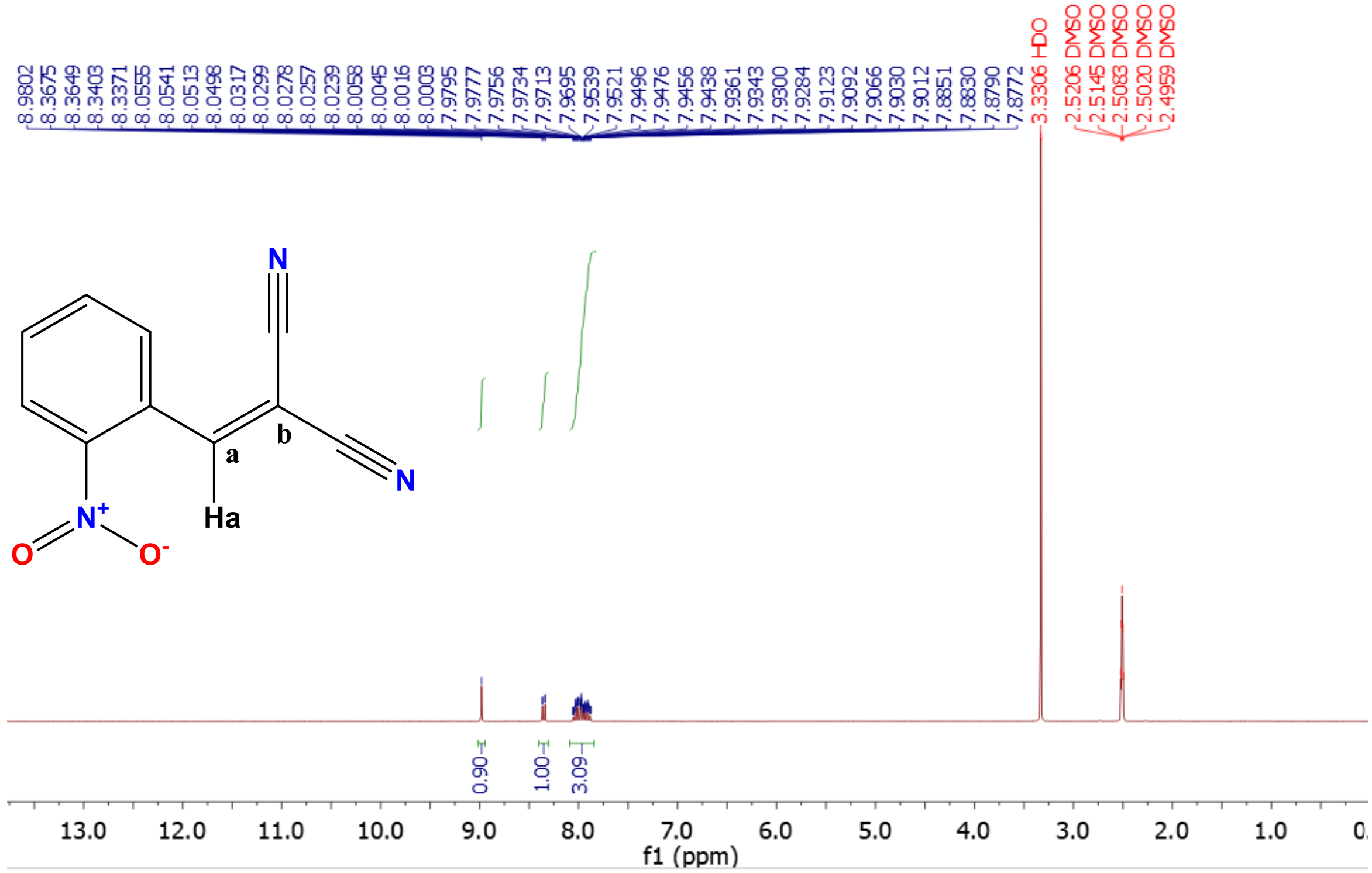

Figure S18. ^{1}H NMR spectrum (300 MHz, **DMSO-d$_6$**) of compound **7**

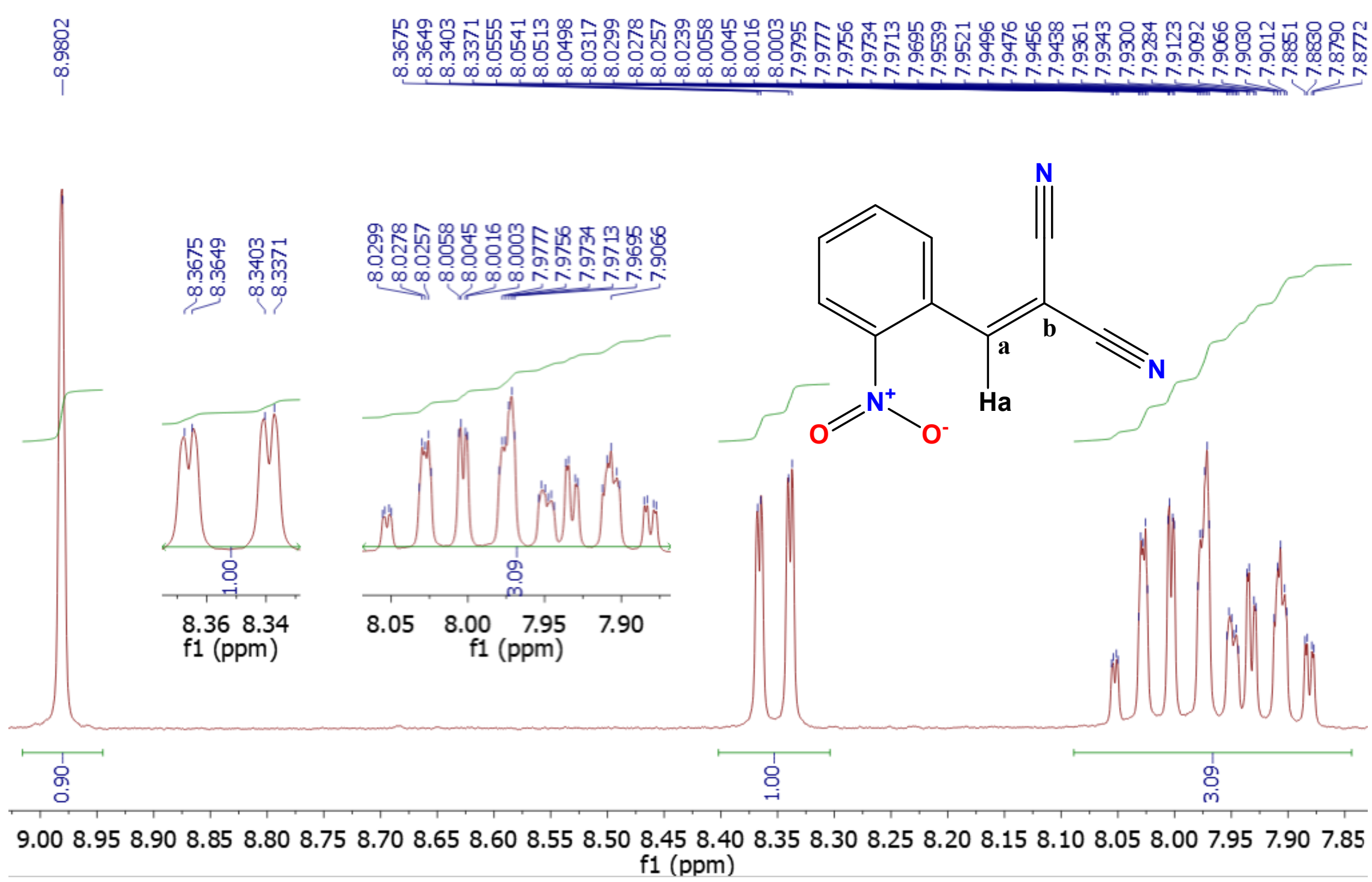

Figure S19. Aromatic enlarged region of ^{1}H NMR spectrum of compound **7**

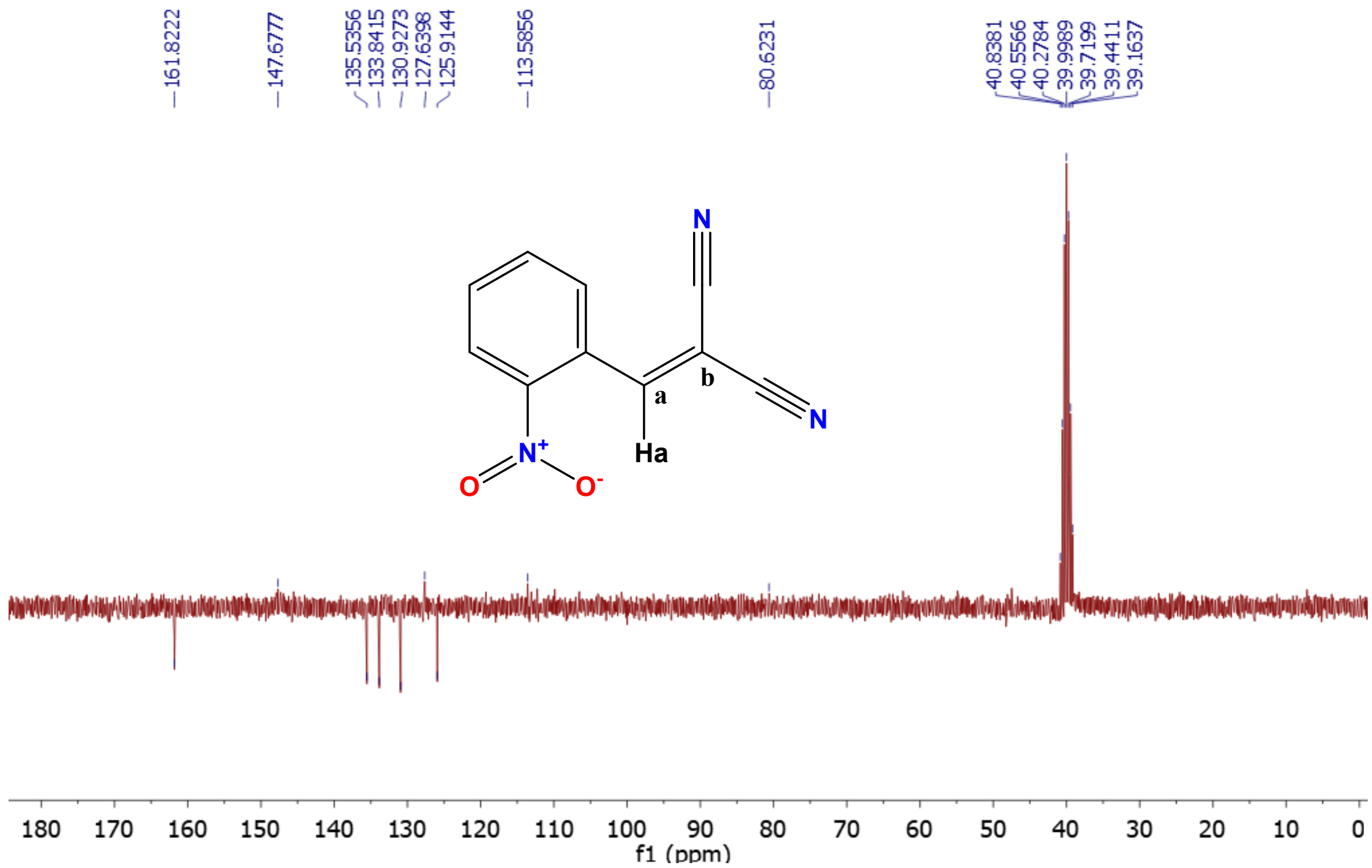

Figure S20. ^{13}C NMR spectrum (75 MHz, **DMSO-d$_6$**) of compound **7**

4.5 ***2-(4-hydroxy-3-methoxybenzylidene)malononitrile* 8:**

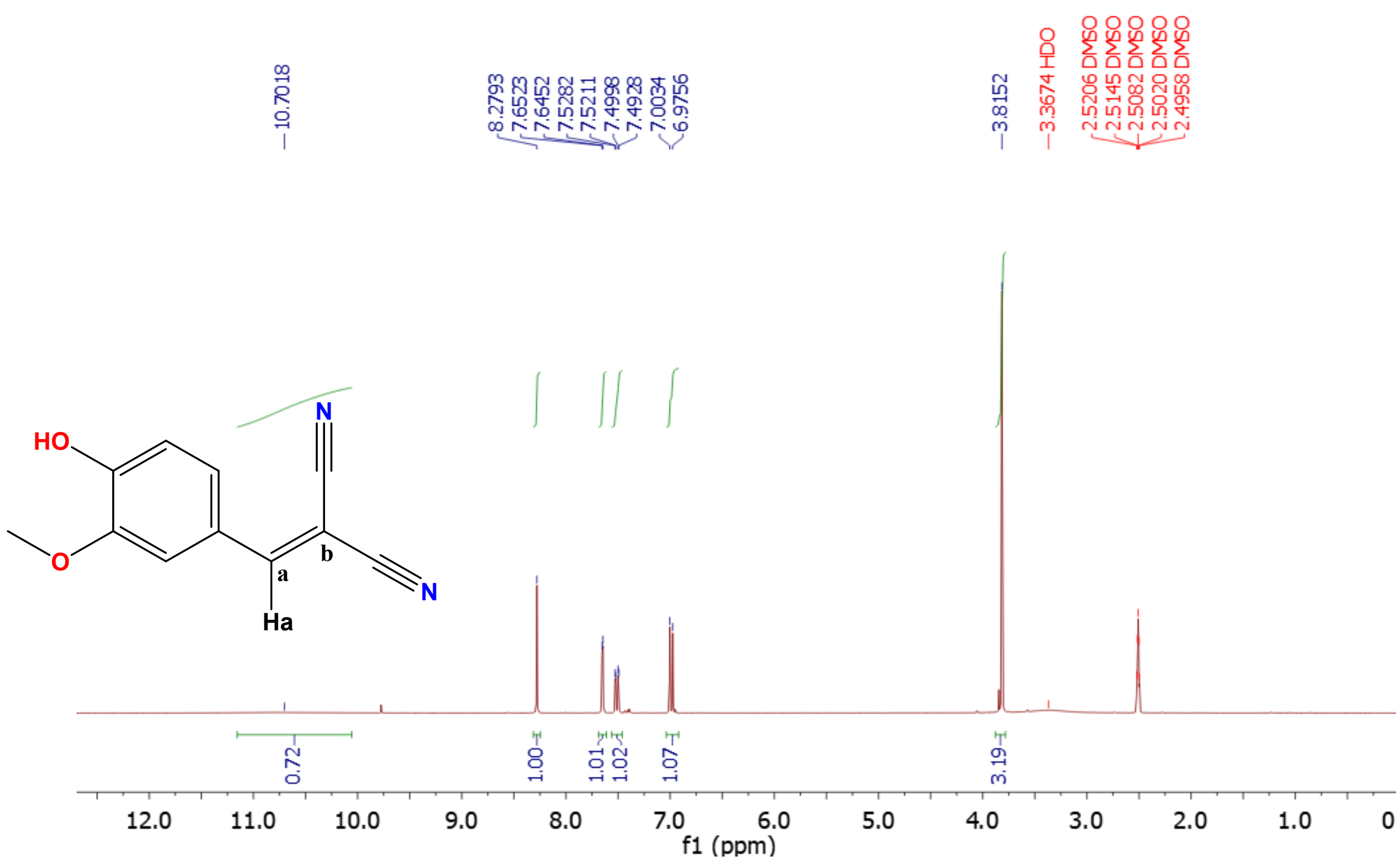

Figure S21. ^{1}H NMR spectrum (300 MHz, **DMSO-d$_6$**) of compound **8**

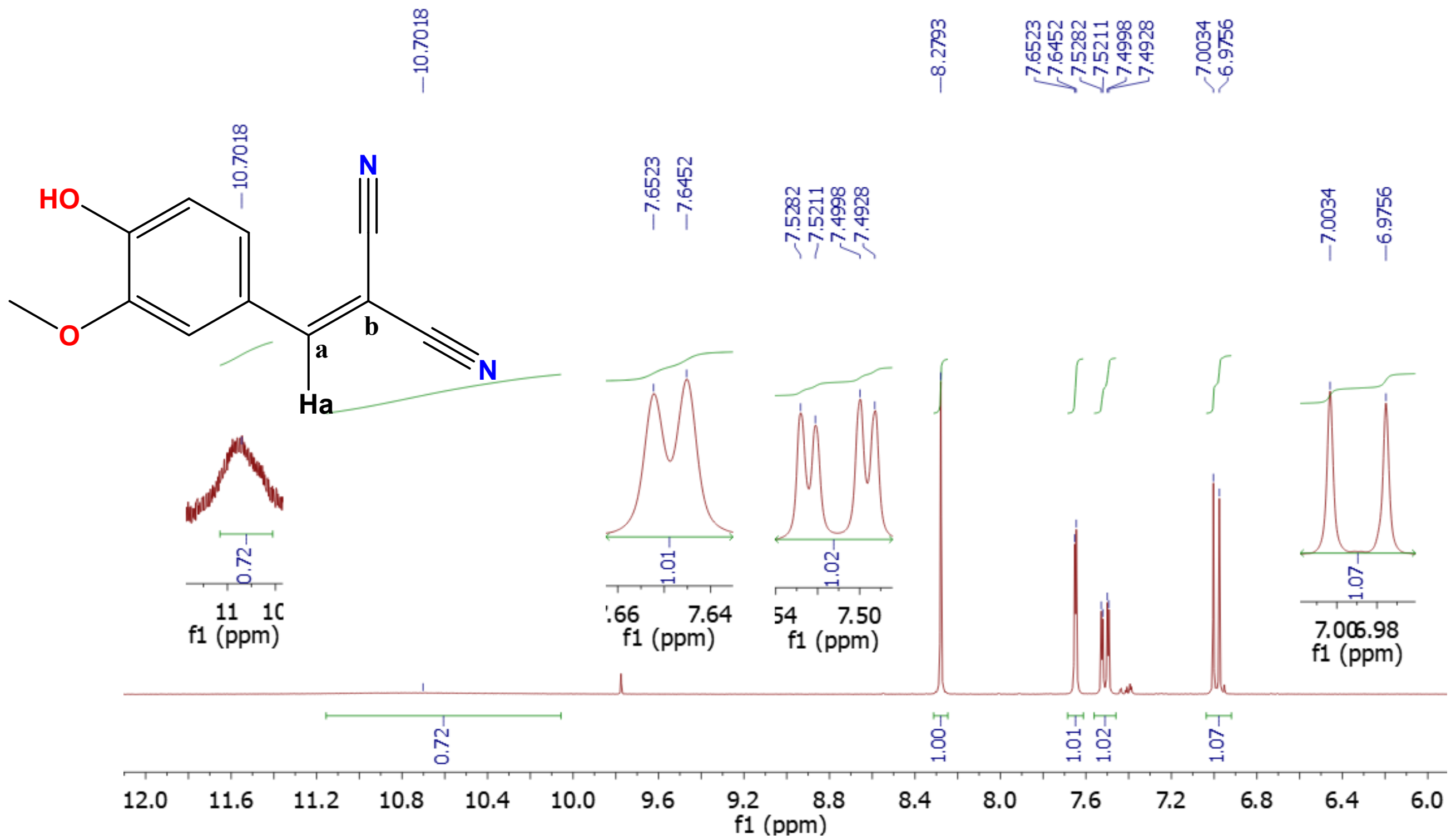

Figure S22. Aromatic enlarged region of ^{1}H NMR spectrum of compound **8**

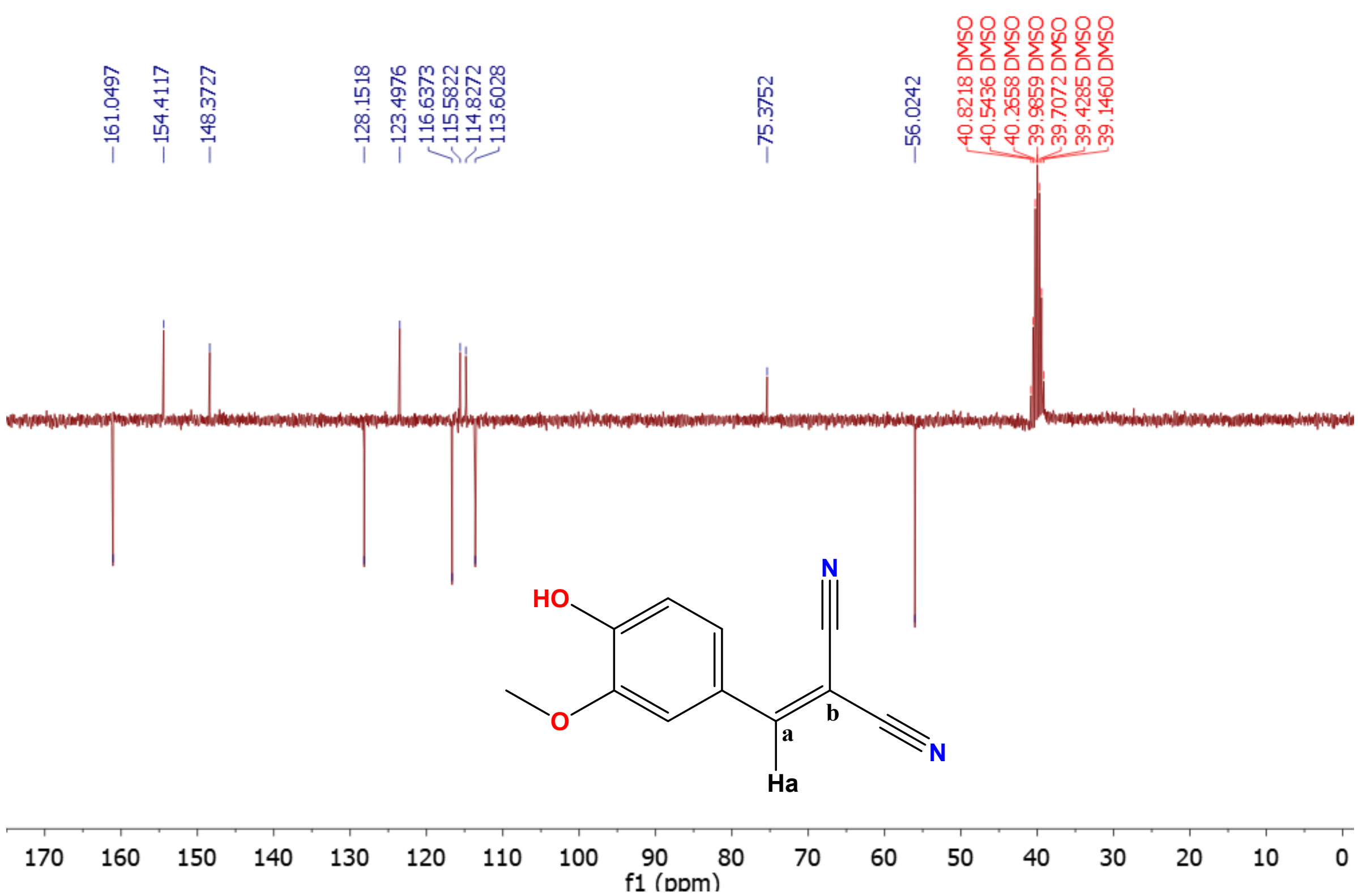

Figure S23. ^{13}C NMR spectrum (75 MHz, **DMSO-d$_6$**) of compound **8**
